# Decoding Catalysis by Terpene Synthases

**DOI:** 10.1021/acscatal.3c03047

**Published:** 2023-09-15

**Authors:** Joshua N. Whitehead, Nicole G. H. Leferink, Linus O. Johannissen, Sam Hay, Nigel S. Scrutton

**Affiliations:** † Manchester Institute of Biotechnology, Department of Chemistry, The University of Manchester, Manchester, M1 7DN, United Kingdom; ‡ Future Biomanufacturing Research Hub (FBRH), Manchester Institute of Biotechnology, Department of Chemistry, The University of Manchester, Manchester, M1 7DN, United Kingdom

**Keywords:** Terpene synthase, Enzyme catalysis, Enzyme mechanism, Carbocation stabilization, Functional plasticity

## Abstract

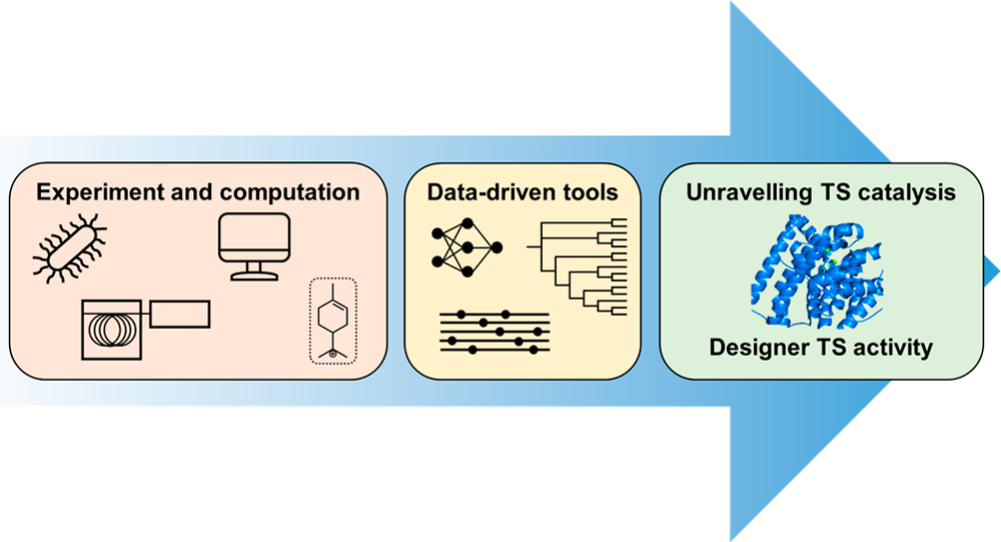

The review by Christianson, published in 2017 on the twentieth anniversary of the emergence of the field, summarizes the foundational discoveries and key advances in terpene synthase/cyclase (TS) biocatalysis (Christianson, D. W. *Chem Rev***2017**, *117* (17), 11570–11648. DOI: 10.1021/acs.chemrev.7b00287). Here, we review the TS literature published since then, bringing the field up to date and looking forward to what could be the near future of TS rational design. Many revealing discoveries have been made in recent years, building on the knowledge and fundamental principles uncovered during those initial two decades of study. We use these to explore TS reaction chemistry and see how a combined experimental and computational approach helps to decipher the complexities of TS catalysis. Revealed are a suite of catalytic motifs which control product outcome in TSs, some obvious, some more subtle. We examine each in detail, using the most recent papers and insights to illustrate how exactly this fascinating class of enzymes takes a single acyclic substrate and turns it into the many thousands of complex terpenoids found in Nature. We then explore some of the recent strategies for TS engineering, including machine learning and other data-driven approaches. From this, rational and predictive engineering of TSs, “designer terpene synthases”, will begin to emerge as a realistic goal.

## 1 Introduction

Terpenes, terpenoids, or isoprenoids are a family of natural products characterized as much by their structural diversity as by their ubiquity. With over 100,000 natural products known, the so-called “terpenome”^1^ represents a third of all the compounds described in the Dictionary of Natural Products.^2^ Terpenoids have industrial value as flavors and fragrances,^3−5^ precursors for pharmaceuticals and bioplastics,^6^ and, more recently, next-generation biofuels.^7,8^ Terpene synthases/cyclases (TSs) help achieve this variety by the seemingly simple conversion of linear, isoprenoid pyrophosphate precursors into highly reactive cationic hydrocarbon intermediates. Upon formation, the cationic intermediate can undergo many changes, ranging from hydride and methyl shifts to cyclization, before the reaction is terminated, typically by hydrogen abstraction or nucleophilic quenching.^9^

TSs carry out some of Nature’s most difficult chemistry, often with more than half of the substrate atoms undergoing a change in bonding or hybridization state by the end of the reaction cascade.^1^ It is the complexity of the reaction profile after abstraction, with its many potential offshoots and quenching mechanisms, that allows for the huge variety of compounds produced by TSs. Two main classes of TSs exist (class I and class II) with different substrate ionization mechanisms and evolutionarily distinct α-helical folds.^10,11^ Class I TSs activate their substrate by metal-dependent ionization of pyrophosphate (PPi), whereas class II TSs use protonation to initiate the reaction cascade.^12^ In class I TSs, the isoprenoid substrate is sequestered by coordination of its PPi group to a trinuclear Mg^2+^ cluster, itself bound by two strongly conserved metal-binding motifs, the aspartate-rich DDxxD/E motif and the NSE/DTE triad.^1^ These motifs are resistant to mutation and are one of the few sequence-level features conserved across class I TSs.^13−15^ Typically, three basic residues (arginines and lysines) also coordinate the PPi, aiding in substrate recognition.^16^ Class I TSs are the most studied and are therefore the main focus of this review.

**Figure 1:**
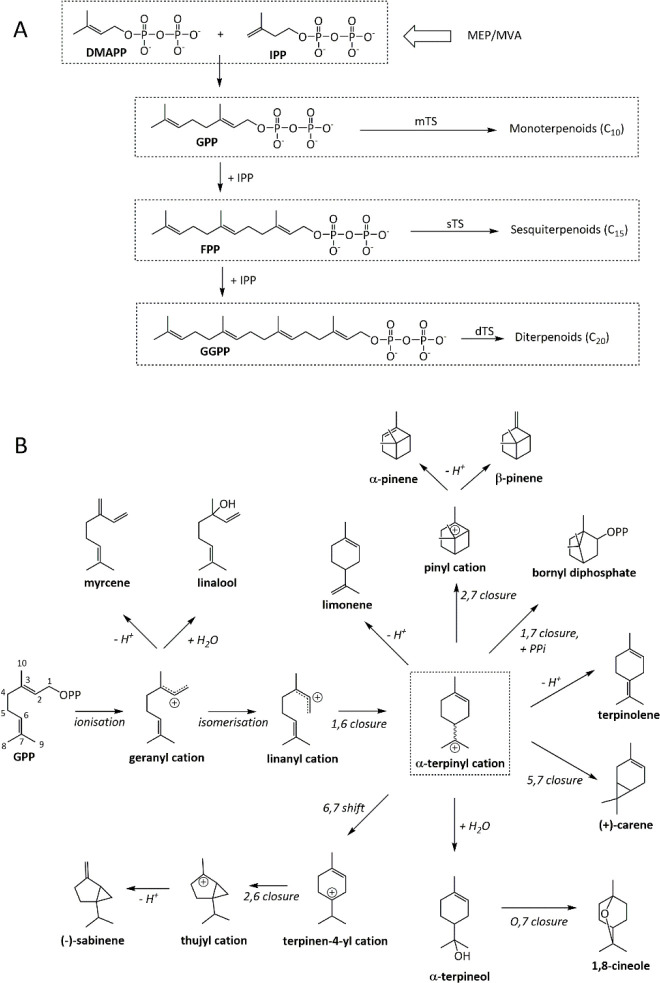
Terpenoids are built from universal precursors. (A) DMAPP and IPP from the MVA or MEP pathways are condensed to give GPP, the precursor of monoterpenoids. Further condensation with IPP yields higher order substrates. (B) Reactions showing some of the different carbon scaffolds achievable from GPP, as discussed in this review. The key α-terpinyl cation is shown in the dashed box and exists as either the (*R*) or (*S*) stereoisomer. Rearrangements are described according to the numbering of GPP.

The substrates of TSs are constructed in a modular fashion from the C_5_ isoprenoid building blocks dimethylallyl pyrophosphate (DMAPP) and isopentenyl pyrophosphate (IPP) (Figure 1). The universally distributed routes to IPP and DMAPP, namely, the mevalonate (MVA) and methylerythritol (MEP) pathways, explain the ubiquity of terpenoids in Nature.^17^ Most bacteria utilize the MEP pathway; eukaryotes possess the MVA pathway, with plants possessing both a cytoplasmic MVA and a plastidial MEP pathway; and archaea utilize an alternative MVA pathway. Head to tail condensation of IPP and DMAPP generates geranyl pyrophosphate (C_10_, GPP), the precursor to monoterpenoids. Condensation of another IPP unit gives farnesyl pyrophosphate (C_15_, FPP), the precursor to sesquiterpenoids, and so on. Head to tail (“1,4”) condensation is required to generate these regular precursors, which will be the focus of this review, although other types of condensation are possible.^1,18^

Terpenoids form a large part of the chemical repertoire of many plants, being used in attack, defense, signaling, and to attract pollinators.^19^ Similarly, it has long been recognized that microbes can make terpenoids.^20^ Geosmin and 2-methylisoborneol are responsible for the characteristic “earthy odor” of newly wet soil (“Petrichor”) and are terpenoid derivatives produced by *Streptomyces* soil bacteria. The constant struggle between different organisms has exerted a selection pressure on the evolution of novel terpenoid scaffolds, and studies have repeatedly shown that changing a small number of active site amino acids is sufficient to change the product profile of a TS.^1,3,21−25^ However, the presence of particular residues in one TS by no means guarantees a similar product in another. Indeed, two TSs that make the same product often have lower sequence identity than with another TS from a related species that makes a completely different product, presenting significant challenges for TS engineering.

TSs have now been discovered and characterized from various kingdoms of life,^8,26−33^ but scaling terpenoid production to levels suitable for industry using heterologous expression of precursor pathways and TSs in microbes (e.g., *E. coli*) presents major roadblocks. One is attributed to the toxicity of the various precursors in the MEP and MVA pathways (and some terpenoids themselves), which inhibit cell growth, even at moderate concentrations.^34^ A second is the inherent complexity and promiscuity of the TS reaction pathways. Much work is being done to address the first issue,^17,35−38^ but this is not the main focus of this review. Instead, we examine how the complexity and promiscuity of TS reaction pathways can be understood and overcome. We survey the recent contributions to the TS literature and examine the various experimental and computational techniques being used to probe and characterize TSs. We then show how these insights enable the fundamental challenges of TS engineering to be tackled.

## 2 Catalytic Motifs in Terpene Synthases

A series of catalytic motifs control the product outcome in TSs, which we will now take in turn. Most TSs make use of more than one motif during terpenoid biosynthesis, sometimes in different stages of the reaction. As such, they ought not to be considered separately when it comes to overall TS chemistry, but for clarity, they are discussed here individually.

### 2.1 The Architecture of the Active Site Determines the Trajectory of the Reaction

**Figure 2:**
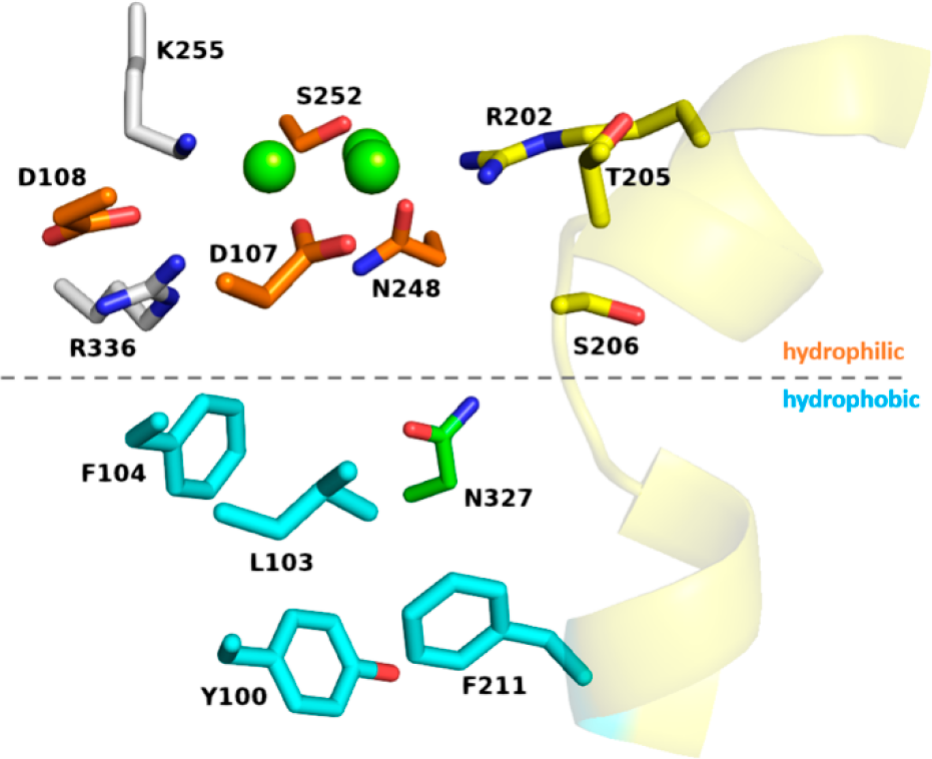
General features of a class I TS active site. The hydrophilic region contains the metal cluster (green spheres), the metal-binding motifs (orange), as well as basic residues (white) and the effector triad (yellow), which sequester the substrate and trigger ionization. The hydrophobic region is defined by nonpolar residues (teal), particularly aromatic residues. Judiciously placed polar residues (green) are essential for catalysis. The catalytically important G-helix with its helix break is shown as a yellow cartoon. This figure is based on the structure of 10-*epi*-cubebol synthase from *Sorangium cellulosum* (PBD: 7ZRN).

Perhaps the most important and intuitive feature of TS catalysis is that the substrate binds in a product- or intermediate-like conformation. The trajectory of the reaction is determined in part by this initial conformation, with substrate rearrangement occurring mostly in an intramolecular fashion (Figure 1). The highly reactive cationic intermediates are susceptible to quenching, so TSs have evolved a binary active site^39^ with a hydrophobic pocket to envelop the hydrocarbon tail, to protect themselves against alkylation (Figure 2). TSs must also exclude bulk solvent, and where nucleophiles are allowed in the active site, this is managed very carefully in ways that will be discussed in section 2.6. Hydrophobic residues, particularly the large aromatic residues phenylalanine, tyrosine, and tryptophan, play an important role in providing the correct active site contour.

TS substrates (GPP, FPP, etc.) are constructed in a modular fashion, where each can be considered a “longer version” of its predecessor (Figure 1). Therefore, the principal method of substrate selection in TSs of different orders (e.g., monoterpene vs sesquiterpene synthases) is simply to modulate active site volume.^40^ This is best illustrated by one of the seminal structures in the field, that of avian FPP synthase (FPPS).^41^ FPPS makes the TS substrate FPP rather than terpenoids themselves, but it contains many of the same motifs and paved the way for understanding of TS chemistry. FPPS catalyzes the condensation of IPP and DMAPP to generate GPP (C_10_), which is subsequently condensed with another IPP molecule to make FPP (C_15_). At this point, the enzyme must ensure FPP is released without further condensation, which it achieves with a “molecular ruler”. Mutagenesis studies of avian FPPS revealed that the side chains of F112 and F133 define the bottom of the active site. Exchange of these residues (F112A and F113S) deepened the active site by almost 6 Å, and incubation with DMAPP and IPP subsequently generated many products of C_20_ and above.^42^ Later studies demonstrated the corollary in that A116W or N114W variants in avian FPPS shifted selectivity strongly toward GPP,^43^ and random mutagenesis of a bacterial FPPS from *Bacillus stearothermophilus* identified several active site residues controlling the distribution between GPP, FPP, and GGPP (C_20_).^44^ Recently, Wang and colleagues solved the crystal structures of three prenyltransferases from *Arabidopsis thaliana* (GGPPS (C_20_), GFPPS (C_25_), and PPPS (C_30_+)—and defined a “three floors” model for determining product chain length. They were then able to predict the catalytic output (carbon chain length) of widely distributed GGPPS-like genes throughout the plant kingdom with 100% success.^45^

This substrate selectivity motif can be harnessed to produce the desired TS substrate. The yeast FPPS Erg20p was engineered by comparison with avian FPPS, with a single mutation (F96W) decreasing the active site volume and reducing the *K*_M_ for GPP 30-fold.^46^ The variant enzyme prefers the IPP + DMAPP reaction over IPP + GPP, improving the GPP:FPP ratio (Figure 1). The authors then further disabled FPPS activity in the protein dimer, before fusing a sabinene (monoterpenoid) synthase with the FPPS double variant to allow immediate sequestering of GPP at the site of production, achieving a remarkable 340-fold improvement in sabinene production.^46^

Substrate selectivity extends to the TSs themselves. Bacterial 1,8-cineole synthase and bacterial linalool/nerolidol synthase (bCinS and bLinS) are a pair of TSs from *Streptomyces clavuligerus*.^47^ Wild-type (WT) bLinS has double the catalytic efficiency with FPP than GPP, making it a sesquiterpene synthase (sTS) with promiscuous monoterpene synthase (mTS) activity.^48^ bCinS is a high-fidelity mTS which not only acts exclusively on GPP, but makes 1,8-cineole at over 95% purity. This is atypical for bacterial TSs, which are usually sTSs that may produce monoterpenes in the bifunctional fashion observed in bLinS.^49^ Comparison of the active site of bLinS with bCinS highlighted residues which might play a role in controlling substrate selectivity.^4^ Several phenylalanine residues were implicated in controlling the size of the active site in bCinS, and the corresponding residues were exchanged in bLinS with the aim of steering product outcome toward linalool. A double variant (L72M-V214L) resulted in no changes in activity (*k*_cat_) toward GPP versus WT, but a 24-fold reduction in activity toward FPP, resulting in an a significantly improved linalool:nerolidol ratio when expressed in engineered *E. coli* for the production of monoterpenoids.^4^ Likewise, when the crystal structure of an ancestral variant of spiroviolene synthase from *Streptomyces violens* was used as a basis for homology modeling of the extant enzyme, a single residue exchange (A224I) was found to dramatically improve the sesquiterpenoid:diterpenoid product ratio, and this isoleucine was inferred as being a more general switch controlling bacterial sTS specificity.^50^

Similar results were reported for a pair of TSs from the Camphor tree *Cinnamomum camphora*. The mTS CiCaMS produces myrcene, whereas CiCaSSy is a sTS that produces santalenes. Remarkably, however, they differ only in 22/553 amino acid residues, implying a very recent evolutionary divergence. Domain swapping and site-directed mutagenesis revealed that only three residue changes (F294M, L403F, L404V) are required in CiCaMS to confer CiCaSSy functionality, with the residues implicated in controlling GPP vs FPP entry.^51^

**Scheme 1:**
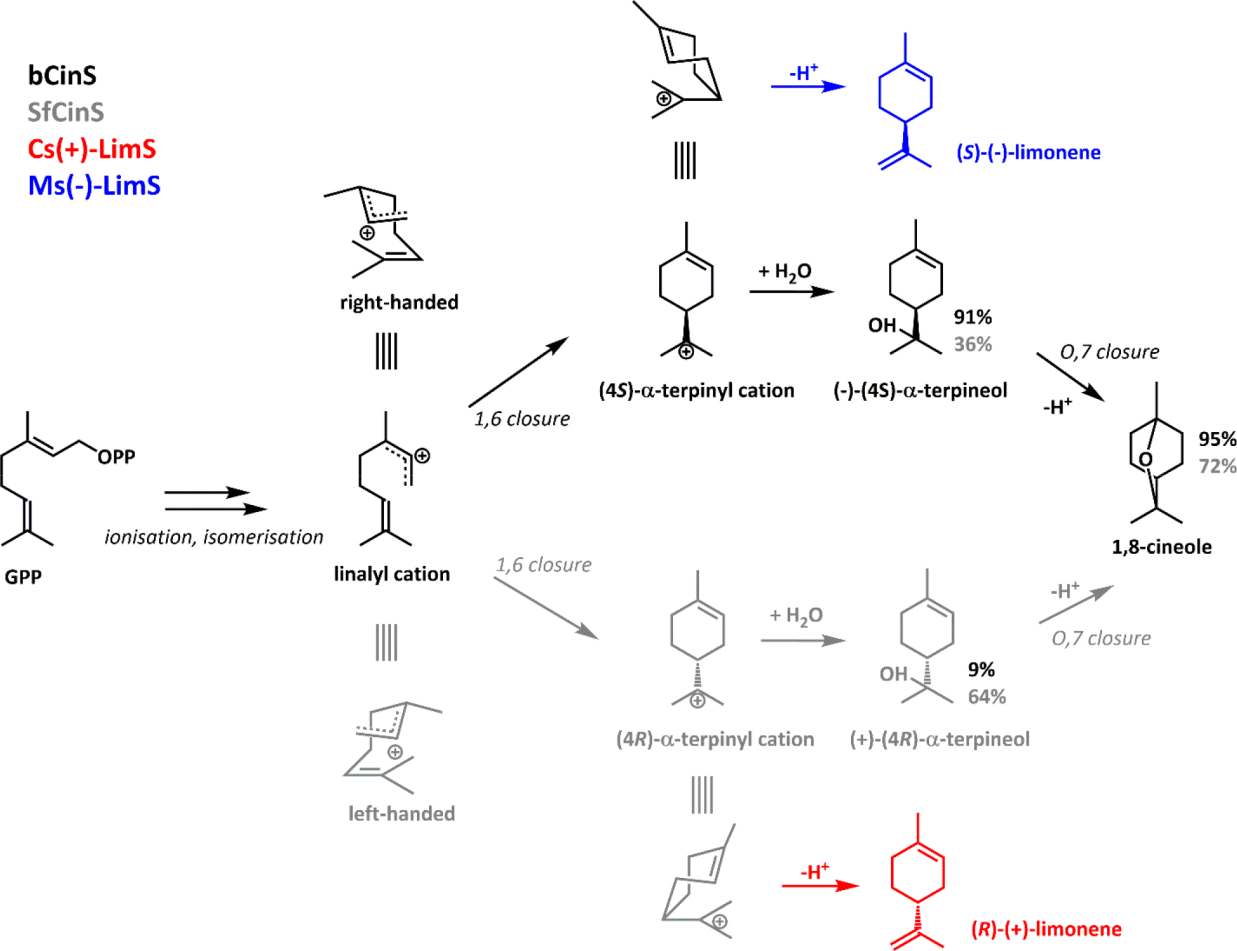
Binding Mode Determines Stereochemistry bCinS (black) and SfCinS (grey) reach the achiral 1,8-cineole by opposite stereochemical routes, depending on the handedness of the binding mode. In bCinS, this mode is enforced more strictly, resulting in higher overall fidelity. The routes to the (−) (blue) and (+) (red) enantiomers of limonene likewise depend on the binding mode.

Perhaps the most emphatic demonstration of the importance of product-like binding in TSs comes from those that make enantiomeric products. Limonene is a cyclic monoterpenoid of industrial interest. It can exist as either the (−) or (+) enantiomer, depending on the stereochemistry of intermediates in the synthesis pathway (Scheme 1). This stereochemistry is thought to depend on the binding orientation. For example, *Mentha spicata* (−)-limonene synthase (Ms(−)-LimS)^52^ binds GPP in a right-handed mode, while *Citrus sinensis* (+)-limonene synthase (Cs(+)-LimS)^53^ binds GPP in a left-handed fashion. Srividya and co-workers showed that a Ms(−)-LimS quadruple variant converts the enzyme from 99% (−) to >80 (+) selectivity with only a marginal change in catalytic activity.^54^

bCinS and plant 1,8-cineole synthase (SfCinS) from *Salvia fruticosa,*(55,56) by contrast, make the achiral product 1,8-cineole, but chiral GC analysis^57^ shows that bCinS catalysis proceeds in the main through (−)-intermediates (91%), whereas SfCinS preferentially makes (+)-intermediates (64%), in accordance with previous labeling studies.^58^ The active site asparagine residues that coordinate the relevant water molecule for α-terpineol formation are located on opposite sides of the active site in bCinS and sfCinS. Exchange of these asparagine residues to alanine destroyed 1,8-cineole formation in both enzymes, further demonstrating that the reaction proceeds in an enantioselective fashion.

Interestingly, bCinS is a high-fidelity mTS, making more than 95% 1,8-cineole from GPP, while its plant analogue SfCinS produces only 67% 1,8-cineole.^47^ It seems that bCinS is able to tightly control the binding orientation of GPP and the formation of a single LPP intermediate (linalyl pyrophosphate, isomerized GPP) and therefore the subsequent α-terpinyl intermediate (Scheme 1).

The results in this section raise important questions about the evolutionary provenance of different orders of TSs. For example, did mTSs evolve from sTSs, or was it the other way round? Or, did mTS functionality evolve from sTSs in bacteria but *vice versa* in plants? Bacterial mTSs show more similarity to bacterial sTSs than they do to plant mTSs.^59^ For example, it has been suggested that bCinS evolved from a higher order (i.e., sesquiterpenoid) TS by contraction of the hydrophobic binding pocket.^47^ Likewise, when Hidden Markov Modeling (HMM) was used to discover and characterize three sTSs from the antibiotic-producing bacterium *Streptomyces chartreusis*, none of these sTSs converted GPP, despite promiscuous mTS activity being fairly common for bacterial sTSs.^60^

Conversely, CiCaSSy shares the greatest sequence similarity with a mTS, a predicted α-terpineol synthase, suggesting it could have evolved from this mTS, which is particularly common to *Cinnamomum.*(51) The difficulty of phylogenetic analysis for TSs has been noted, prompting different approaches. Recently, 262 plant sTSs were collated and divided into groups according to the presumed parent cation of their major product, yielding more distinct groups than conventional phylogenetic analysis.^61^ The authors note that TSs with dual-substrate activity (GPP and FPP) very often make sesquiterpenoids derived from the bisabolyl parent cation, the result of 1,6 FPP cyclization. The bisabolyl cation possesses a cyclized “head” and uncyclized “tail”, and may be considered almost as a cyclic monoterpenoid with an “isoprenoid extension”. Expansion of the active site could confer the requisite sTS activity on an existing mTS, as demonstrated in CiCaMS. However, CiCaSSy produces several bi- and tricyclic sesquiterpenes, unlike myrcene production in CiCaMS, which is rather simple. This might suggest the opposite conclusion, that CiCaMS is a loss-of-function sTS.^51^ Both arguments find support, and at present, one cannot say with any certainty which class of enzymes came first, although answering this question will surely yield important insights for the rational design of TSs.

### 2.2 Intrinsic Reactivity and Negative Catalysis: How Much Do TSs Actually Do?

With the occasional exception of water addition (section 2.6) the rearrangement of the TS substrate occurs intramolecularly. TSs handle highly reactive intermediates but regularly manage to generate a single product at significant purity. Despite this, TSs are often characterized simply as hydrophobic molds, taking little part in the reaction other than providing a template for the already highly favored rearrangement of the substrate. Is this a correct interpretation?

It is certainly true that the highly reactive nature of the carbocationic intermediate provides the driving force for reaction. This is observed in gas phase calculations,^62−68^ where catalytically relevant rearrangements occur spontaneously. Reactivity in the gas phase (i.e., in the absence of the enzyme *and* water) therefore represents the reactivity built into the molecule itself, its *intrinsic reactivity*. Since solvent is presumed to temper the reactivity of the substrate (relative to the gas phase), Tantillo raises the possibility that the role of TSs (and other enzymes) may in fact be to allow intrinsic reactivity to be expressed, rather than suppressed.^69^

**Scheme 2:**
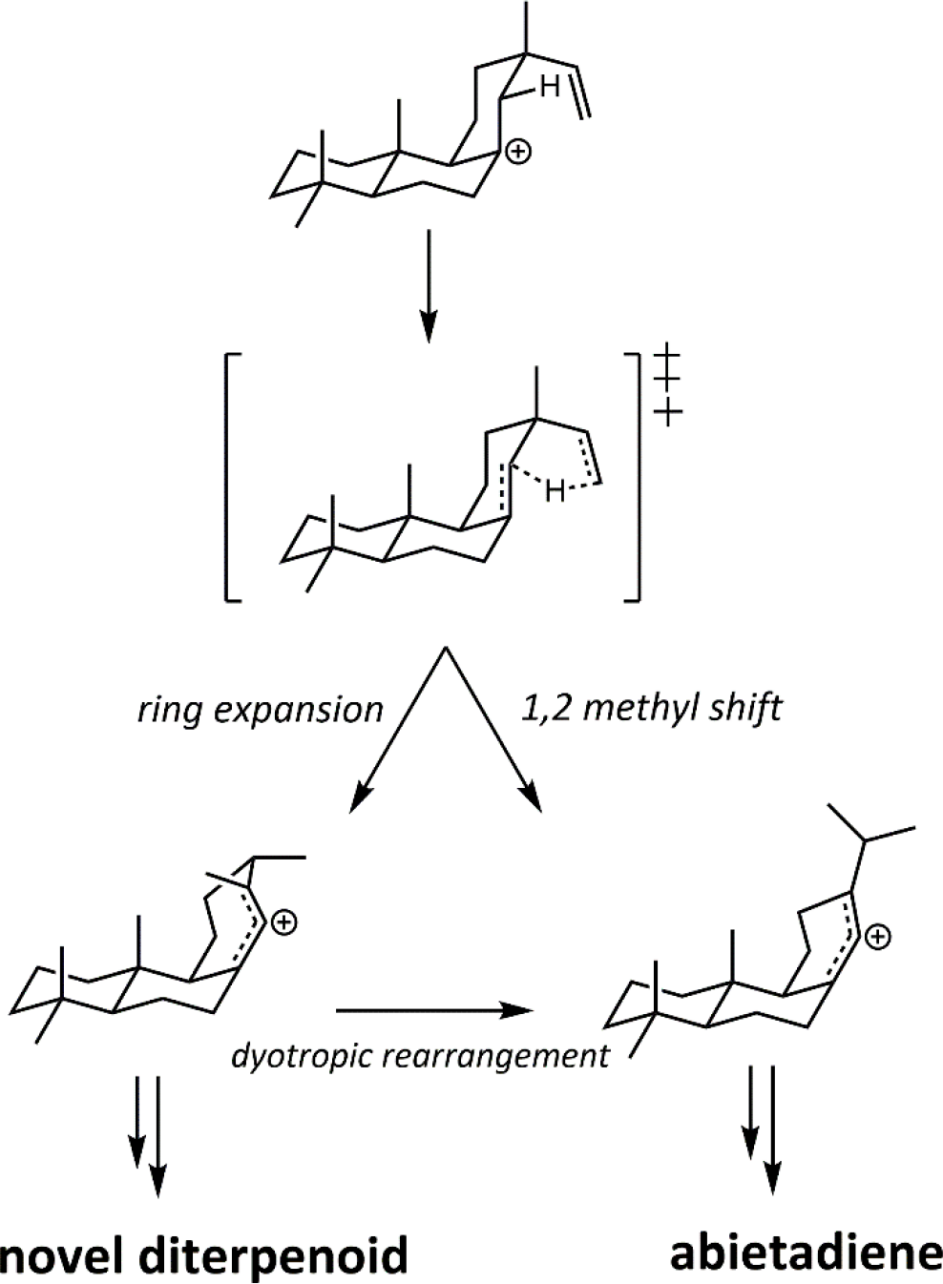
Intrinsic Reactivity and Enzymatic Intervention in Abietadiene Biosynthesis After the transition state shown the reaction coordinate bifurcates. Gas phase calculations show an inherent dynamic preference for the 1,2 methyl shift (intrinsic reactivity), although substantial amounts of the ring-expanded product are still observed. Abietadiene synthase discourages this natural dynamic tendency and achieves 95% abietadiene.

That said, enzymes clearly demonstrate selectivity above and beyond that observed in the gas phase. For example, on the way to the diterpenoid abietadiene there is a key transition state, after which the reaction pathway bifurcates, with no energy barrier for one of two pathways: one leads to the precursor of abietadiene by 1,2 methyl shift and the other via ring expansion to a diterpenoid not observed in Nature (Scheme 2). Nonetheless, formation of the abietadiene precursor is preferred in the gas phase by a ratio of 1.1–1.7:1 (depending on the model system and level of theory used). This implies there is a preference for abietadiene formation even in the absence of the enzyme, although there is still a significant amount of the ring-expanded side product. The enzyme, by contrast, achieves 95% abietadiene. Likewise, gas phase calculations of the carbocation rearrangements required for miltiradiene formation suggest that ∼50% of trajectories should lead to a rearranged product not observed in miltiradiene synthase. These examples and others suggest that TSs intervene to achieve a specificity far greater than intrinsic reactivity alone.^70−78^

Abietadiene synthase neatly demonstrates the relationship between the isoprenoid substrates and TSs in that intrinsic reactivity is the driving force for reaction, and the natural dynamic tendency of the substrate is harnessed as a starting point, with subtle interventions made enzymatically to further tune the product or improve selectivity.

**Figure 3:**
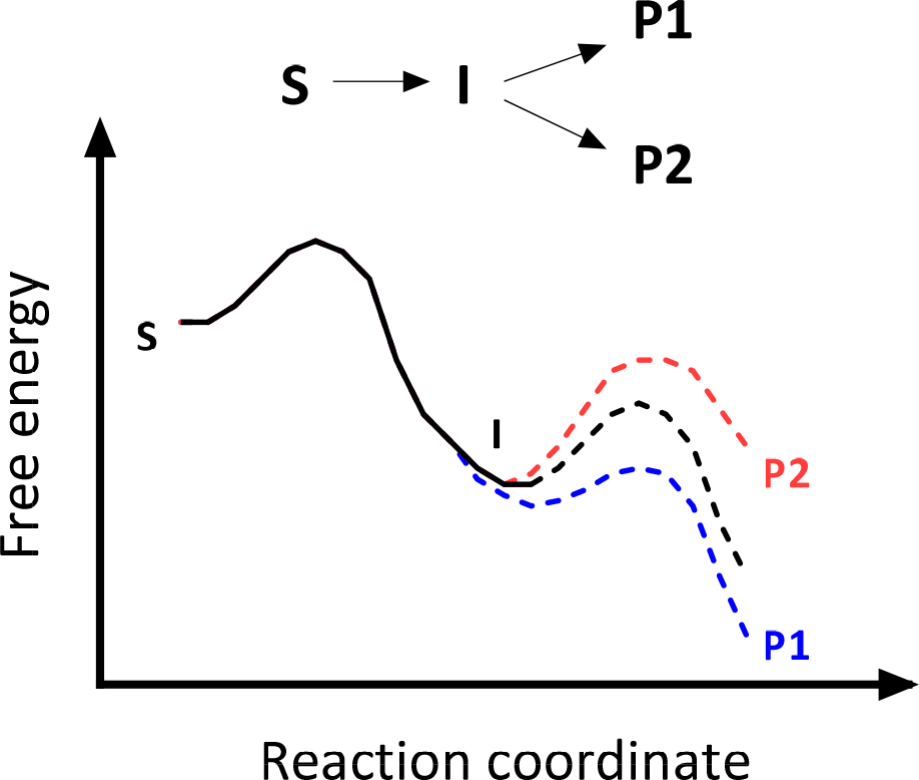
Positive and negative catalysis. In this simple reaction, the enzyme can either stabilize formation of the desired product (positive catalysis, blue) or destabilize formation of an undesired product (negative catalysis, red).^79^

In theory, an enzyme can privilege a particular reaction coordinate either by (1) lowering the energy of the relevant transition state or (2) raising the energy of transition states along the alternative pathways (Figure 3). This is “positive” and “negative” catalysis,^79^ respectively. An amino acid residue therefore makes a positive or negative catalytic contribution depending on whether it enhances the turnover number *k*_cat_ (1) or improves the ratio of desired:undesired products (2). It is often difficult to assign a positive or negative catalytic effect to a particular residue, since these effects are rarely totally separate. Moreover, certain experimental set-ups may not detect a particular effect: for example, substrate consumption assays would measure the effect of mutation on *k*_cat_, but not on the resulting product ratio. Nonetheless, the evidence for a negative catalytic effect is growing, and TSs represent a prescient example.^39,79,80^

For example, taxadiene synthase from *Taxus brevifola* (TXS) makes 93% of the diterpenoid taxadiene from GGPP. The W753H variant reduces activity by 50% but completely redirects the reaction to a side product, (−)-(*R*)-cembrene A, which is not detected in the WT, suggesting that W753 exhibits a mostly negative catalytic effect. It is possible that W753H could lower the transition state of the alternative reaction (positive catalytic effect) by acting as a base, but we would expect to see a concomitant increase in *k*_cat_. Even more dramatically, V584L retains 90% activity, but formation of the side product verticillia-3,7,12(13)-triene leaps from 0.8% to 84%. TXS, therefore, apparently makes use of negative catalysis to prevent the formation of unwanted side products. There is a balance to be achieved, however: the Y89E variant is actually *more* specific than the WT, but at the expense of reduced activity (2.8% of WT). It could be that TXS has made a trade-off between specificity and activity at this position.^81^

**Scheme 3:**
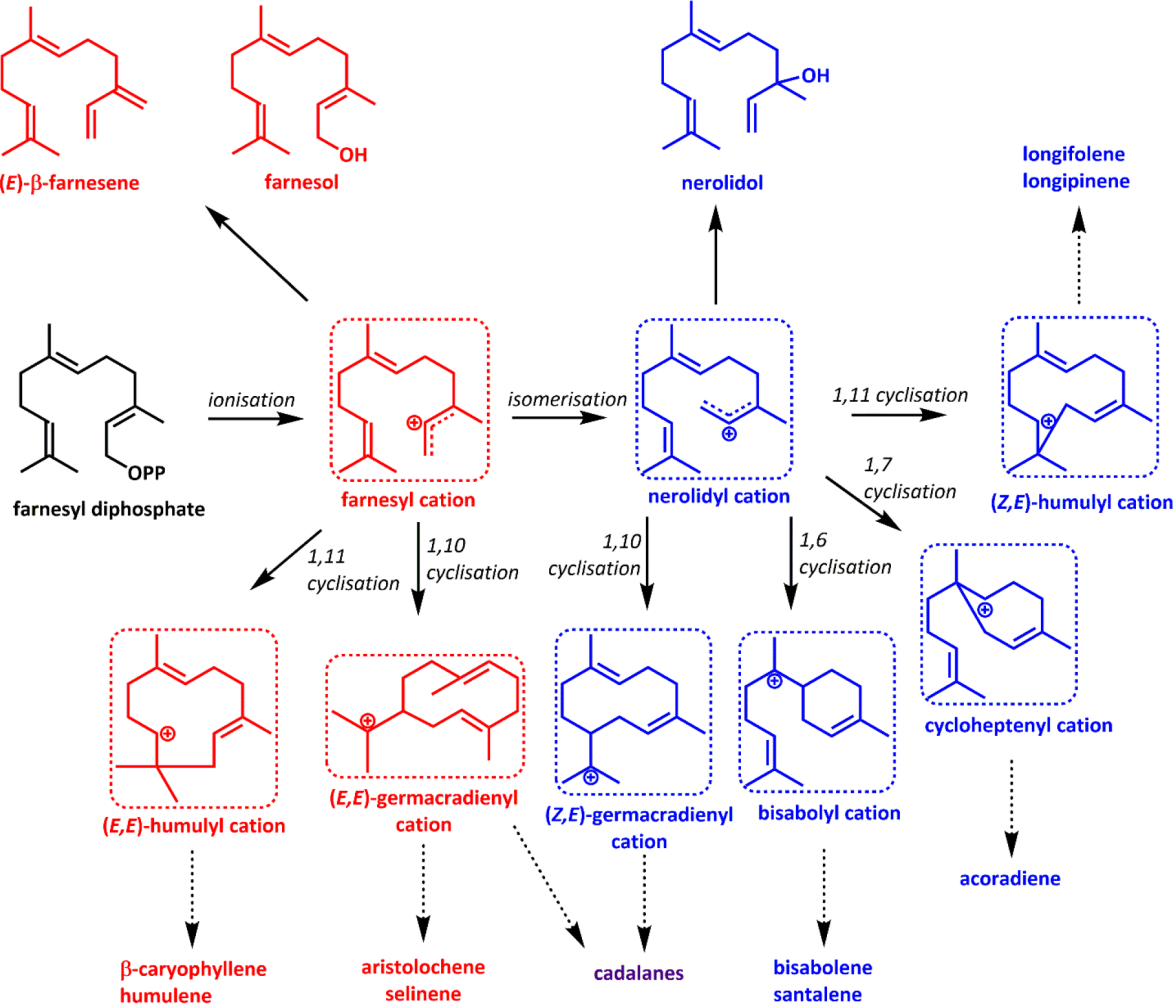
Formation of Cyclic Sesquiterpenoids All sTS-catalysed reactions start with the ionization of FPP. The resulting farnesyl cation is converted to the nerolidyl cation (via nerolidyl pyrophosphate, not shown) following isomerization. Both the farnesyl and nerolidyl cations can produce linear products or cyclic intermediates. Products derived from the farnesyl cation are shown in red, those from the nerolidyl cation in blue, and those from both in purple. This figure was reprinted as a modified version from ref (92) with permission under the creative commons CC-BY 4.0 license.

Trichodiene synthase (TDS) provides another example. TDS produces the sesquiterpenoid trichodiene from FPP at 84% purity, the parent compound in the trichothecane family of mycotoxins and antibiotics.^82,83^ When Dixit and colleagues^84^ performed quantum mechanics/molecular mechanics (QM/MM) and QM calculations on the enzymatic and gas phase reactions, respectively, they found that in the gas phase ring closure of the farnesyl cation to yield the key bisabolyl cation (Scheme 3) is exergonic (−18 kcal/mol) and proceeds without a barrier. In the enzyme, however, the energy of the bisabolyl cation is raised by ∼20 kcal/mol relative to the gas phase, resulting from the loss of electrostatic interactions in the farnesyl cation–PPi ion pair. This negative catalytic effect results in a concerted cyclization cascade which significantly reduces the opportunity for premature quenching of the bisabolyl cation. This intermediate is partially stabilized, however, by a novel sulfur−π interaction between M73 of the enzyme and C7 of the substrate that acts as an anchor, preventing substrate tumbling and premature quenching. This positive catalytic interaction also reduces the reactivity of the intermediate, permitting subsequent catalytic steps. TDS therefore exploits all three features described in this section: first, it permits the highly reactive substrate to rearrange, for the most part, in the manner observed in the gas phase, owing to its intrinsic reactivity. It employs negative catalysis to increase the energy of a key branching point (the bisabolyl cation), from which multiple side products could be derived. Lastly, the novel Met–substrate interaction stabilizes the same intermediate and helps lock it into a productive conformation for the subsequent stages of the reaction–positive catalysis with respect to trichodiene synthesis.^84^ Similar results were reported for cyclooctat-9-en-7-ol synthase (CotB2), which utilizes intrinsic reactivity while also employing a mix of cation−π, dipole–cation, and ion-pair interactions which do not serve to enhance the rate of the overall reaction but guide the reaction along the correct pathway.^85^ In answer to the question posed at the beginning of this section, therefore, we claim that TSs do a great deal, although often in ways that are very subtle and difficult to notice at first glance.

### 2.3 Cyclization

Cyclization is the signature step in TS reactions. After substrate ionization, isomerization about the new C2,3 single bond from a *trans* to *cis* configuration yields the appropriate precyclic conformation for monoterpenoid synthesis and expands cyclization potential for the higher order substrates. In monoterpenoid synthesis, C1,6 cyclization of the linalyl cation (ionized linalyl pyrophosphate) yields the key α-terpinyl cation, the precursor of hundreds of monoterpenoids with a variety of final scaffolds (Figure 1). The additional isoprenoid unit in FPP allows for even more variety: cyclization is possible via C1,6, C1,7, C1,10, and C1,11 bond formation, and multiple cyclization steps are possible (Scheme 3). Higher-order terpenoids are the precursors to many important biological molecules, from naturally produced vitamins and steroids^68,86−88^ to drugs such as the antimalarial artemisinin^89^ and the chemotherapeutic drug Taxol,^90^ both produced by engineered microbes. A cryptic plant TS capable of producing macrocyclic 14- and 18-membered terpenoids was also recently characterized whose products showed significant immunosuppressive activity.^91^

The mechanism of isomerization has been established elsewhere^1,93−95^ and will not be discussed in detail here. However, the isomerized substrate itself warrants closer inspection. The existence of LPP (isomerized GPP) was suspected for cyclic monoterpenoid synthesis, since the C1–C6 bond distance and orientation in GPP is not appropriate for cyclization, and the barrier to bond rotation in the allylic cation is presumed to be too high.^1^ Since isomerization is required for the correct precyclic conformation of GPP, and many TSs will accept LPP as an alternative substrate, the existence of the LPP intermediate is strongly implied. Improved catalytic efficiency with isomerized substrates also suggests that ionization and isomerization are the slowest chemical steps in TS-catalyzed reactions.^96^ In light of the discussion about negative catalysis, one might say that the most conventional catalytic role played by TSs (speeding up a reaction) is the ionization/isomerization step.

The existence of LPP has now been supported definitively by kinetic analysis with fluorinated analogues.^97^ In Cs(+)-LimS, steady-state kinetics with LPP resulted in burst-phase kinetics but not with GPP, implying the isomerization is rate-limiting. Studies with 2-fluoro-GPP, which blocks isomerization, have shown that the reaction will not proceed with this substrate. Morehouse and co-workers synthesized an alternative analogue, 8,9-difluoro-GPP, which ought to allow isomerization of GPP to LPP, but prevent the subsequent cyclization to the α-terpinyl cation, the precursor to limonene (Figure 1). Indeed, the reaction with this substrate was 64 times slower than with GPP, despite having a similar *K*_M_, implying a stepwise reaction via LPP. Furthermore, soaking apo-Cs(+)-LimS with 8,9-difluoro-GPP yielded crystals with 8,9-difluoro-LPP, demonstrating isomerization has occurred. Intriguingly, however, it should be noted that LPP is observed in the extended conformation, not the precyclic one.^97^

This is consistent with results reported by the same group for Ms(−)-LimS. Molecular dynamics (MD) simulations of Ms(−)-LimS complexed with either GPP or LPP showed that the extended GPP complex is stabilized within 5 ns, but the helical LPP complex is not stabilized after 10 ns. In both simulations, H579 inserts into an important position, but for GPP this happens more quickly, and for LPP requires additional conformational change (movement toward Y573). The authors note that Y573 equivalents are totally conserved in cyclic TSs, but are more variable in linear TSs, suggesting a role in achieving the correct conformation of the substrate.^98^ Interestingly, and consistent with the findings for Cs-(+)-LimS, when the authors fed neryl pyrophosphate (NPP, *cis*-isomer of GPP) to the Ms-(−)-LimS Y573 variants, they could not make limonene at WT levels, implying that isomerization is not the only role this residue is playing. It has been noted elsewhere that the product profile can change in mTSs by using NPP versus GPP.^99−102^ The different binding modes clearly impact catalysis, even for enzymes that are known to proceed via isomerization. To further complicate matters, when the sTS pentalenene synthase (PS) was incubated with difluoro-FPP (which should block cyclization only), not even ionization products were observed, suggesting that in this enzyme the ionization step may proceed via direct displacement due to ring closure (i.e., as part of a concerted, rather than a stepwise mechanism).^103^ The difference between the S_N_1 and S_N_2 character of this step in different TSs has also been raised elsewhere^104^ and is a significant finding given that ionization is assumed to the slowest chemical step in TSs.^84^

All of this should be considered when drawing conclusions from crystal structures or models derived from them (e.g., homology models). TSs undergo an induced-fit rearrangement upon substrate sequestration, with Baer and colleagues for example showing that the effector residue of selinadiene synthase moves more than 5 Å upon FPP binding.^93^ Also, as discussed, substrate flexibility and orientation are key factors in catalysis by TSs. It is therefore important to determine whether an enzyme or enzyme–substrate complex is in a conformation relevant to catalysis. Interaction of the substrate with the motifs described in this review, for example, the effector triad and the metal-binding motifs, might serve as a good starting point.

In a natural setting, terpenoids have diverse roles ranging from antipest compounds and antibiotics to signaling molecules and pollinator-attracting compounds. The evolutionary benefits of an expanded chemical space become abundantly clear in this context, and cyclization is the major way of achieving this. There is therefore considerable interest in understanding how this came about, not least because this knowledge will surely aid future engineering efforts. (*E*)-β-Farnesene synthase (BFS) and amorpha-4,11-diene synthase (ADS) from *Artemisia annua* produce a linear and cyclic sesquiterpenoid from FPP, respectively. BFS perhaps represents the simplest type of TS: ionization of FPP followed by deprotonation yields (*E*)-β-farnesene (Scheme 3). ADS, by contrast, requires ionization, isomerization, and two cyclizations, and yet the two sequences share 49% similarity, high by the standards of TSs. Salmon and co-workers discovered that a single mutation (Y402L) was able to confer cyclization activity onto BFS.^105^ Variants containing Y402L, but with WT-like BFS activity, helped reveal an epistatic network controlling cyclization: Y402 induces cyclization; V467G suppresses it (i.e., in the Y402L-V467G double variant); and Y430A reinduces it (i.e., Y402L–Y467–Y430A), but Y430A is not sufficient to induce cyclization on its own. Remarkably, when this epistatic network was tested in an analogous BFS from *Citrus juno* it also induced cyclization. As discussed, cyclization depends on isomerization. Salmon and colleagues^105^ describe this step as “unlocking” the unproductive *trans* conformation FPP to yield the *cis* form, oriented correctly to cyclize to the bisabolyl cation. In BFS this does not occur, deprotonation instead terminates the reaction. Y402L must therefore prevent or delay this deprotonation as well as permit the steric rearrangement required for isomerization. Phylogenetic analysis reveals tyrosine is the ancestral state of residue 402, implying the Y402L mutation was a recent event in *A. annua*. The presence of this substitution in dedicated α-bisabolol synthases suggests it was a dominant factor in the emergence of cyclization in these two types of TSs. The epistatic network that prevents reversion to BFS activity by reciprocal L402Y mutation represents a subsequent tuning of activity toward high-fidelity cyclization.

In ADS itself Abdallah and colleagues identified other residues important for achieving the two cyclization steps, and a mutability landscape revealed variants with improved catalytic efficiency and kinetics.^106^ H448A exhibited a 4-fold increase in catalytic efficiency, and the double variant T399S/H448A improved *k*_cat_ 5-fold, at least with respect to PPi release. Full product profiles are required to determine what effect this variant has on enzyme specificity, but if unchanged this variant could be used for enhanced production of amorphadiene, the precursor to the important antimalarial artemisinin.

The importance of tyrosine in modulating cyclization is further illustrated by a mushroom linalool synthase from *Agrocybe pediades* (ApLS). This enzyme was originally reported by Zhang and co-workers to reach linalool titers of >600 mg/L at exceptional enantiopurity and with no nerolidol formation when expressed in *E. coli* engineered for terpenoid production.^26^ Recent success with obtaining a crystal structure revealed that product selectivity in this enzyme depends on a key tyrosine residue.^107^ Exchange of Y299 is sufficient to abolish the selectivity of ApLS toward GPP and acyclic products. Modeling based on the solved crystal structure, the first of a fungal mTS, revealed a greater affinity of ApLS for GPP than FPP due to torsional strain on the hydrocarbon tail of FPP. ApLS possesses a trio of residues, Y299–S184–M77, which create a constriction in the substrate tunnel, precluding the longer substrate. Comparison with ApLNS (a bifunctional fungal linalool/nerolidol synthase) reveals that ApLNS possesses an isoleucine at position 61, rather than a valine in ApLS, which noticeably alters the position of Y299 and therefore the size of this substrate channel. Notably, M77 in ApLS corresponds to L72M in bLinS, a variant which helped preclude FPP and improve the linalool:nerolidol ratio.^4^ Lastly, comparison of ApLS with two other microbial TSs, bCinS, and *Sorangium cellulosum* 10-*epi*-cubebol synthase (ScCubS), and sequence alignment with the 185 most similar fungal TSs revealed that the majority of these possess asparagine at position 299 (equivalent) and produce cyclic products. Those which make acyclic products, however, possess tyrosine. This tyrosine/asparagine “switch” may therefore represent an attractive target for rational engineering of TSs.^107^

Identifying these synergistic effects is crucial for any rational engineering effort since these epistatic changes have been shown to apply to other TSs. We noted above how the epistatic network revealed in BFS induced cyclization in an analogous BFS from *C. juno*. A similar result was reported for a labdane-related diterpene synthase (LRDS). LRDSs are clearly derived from *ent*-kaurene synthase (required by all land plants for phytohormone biosynthesis) and are therefore often called kaurene synthase-like (KSL). Two alleles of a rice KSL (*Oryza sativa*) were found in a previous study to quench the reaction after 3 cyclizations instead of the 4 needed to make the final kaurene product by mutation of single residue (I664T).^108^ Further investigation by Peters and colleagues identified a second residue (V661) that tunes product outcome by exerting an epistatic effect in conjunction with the previously identified isoleucine.^109^

**Figure 4:**
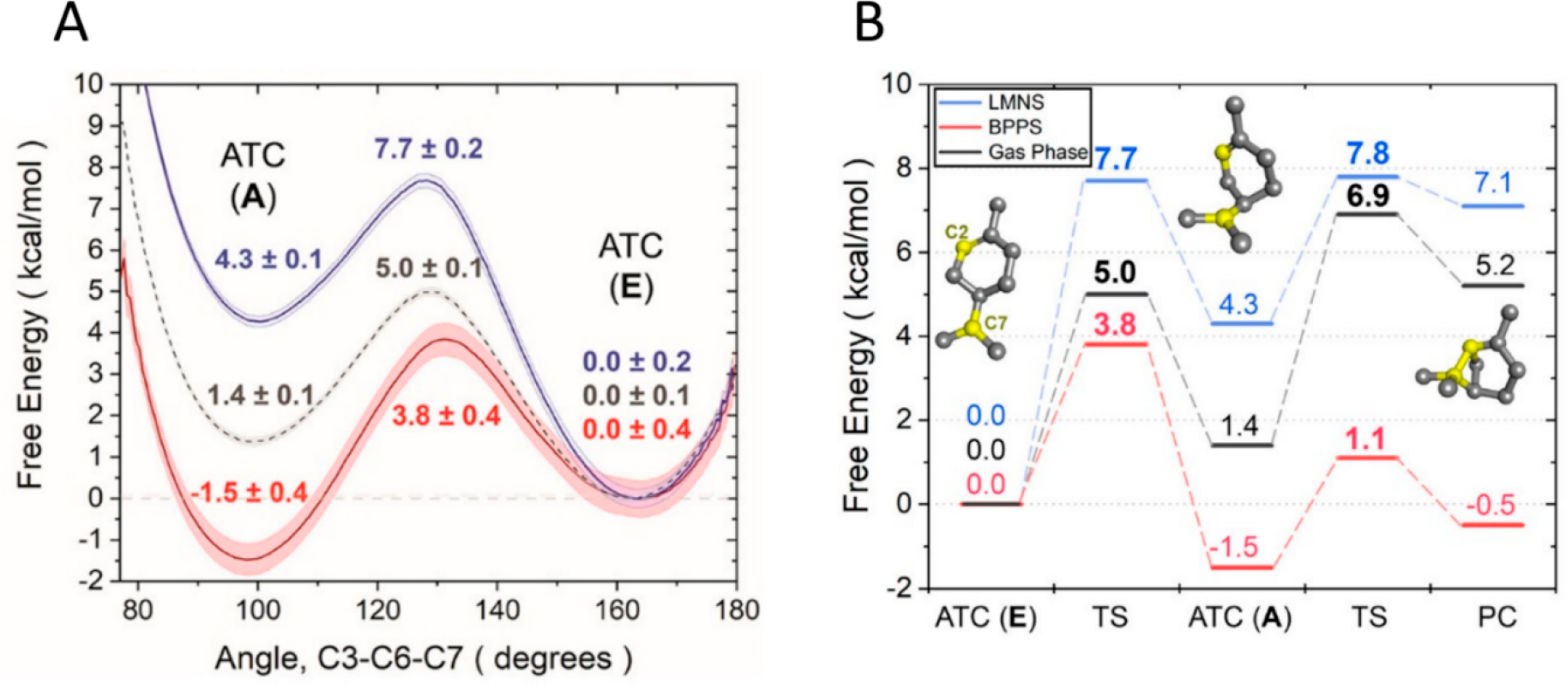
Free energy calculations suggest that the α-terpinyl cation (ATC) assumes different conformations in Ms(−)-LimS and BPPS. (A) Free energy profile for the interconversion of the axial and equatorial α-terpinyl cation in Ms(−)-LimS (blue), BPPS (red), and the gas phase (gray dash). Each enzyme shows an opposite preference. (B) Free energy profile for formation of the pinyl cation (PC) in Ms(−)-LimS, BPPS, and the gas phase (same color scheme). The reaction is more favored and facile in BPPS. Panels A and B were reprinted without modifications from ref (110) with permission under the creative commons CC-BY-NC-ND 4.0 license.

TSs must carry out a specific number of cyclization steps depending on the desired product. This is particularly important for the longer substrates of C_15_ and above, but even GPP (C_10_) possesses sufficient functionality for two cyclizations. The mechanistic basis for mono- or bicyclic selectivity was explored in detail for the model mTSs Ms(−)-LimS and (+)-bornyl diphosphate synthase (BPPS).^110^ Limonene is monocyclic, and bornyl diphosphate is bicyclic. Both proceed via the key α-terpinyl intermediate, but while limonene results from direct deprotonation, bornyl diphosphate requires a second ring closure of the α-terpinyl intermediate to the pinyl cation (Figure 1). This second cyclization depends on the appropriate orientation of the isoprenoid tail: it must be axial with respect to the first ring, whereas in limonene synthesis it is presumed to be held equatorially. Kim and co-workers^110^ have demonstrated that the α-terpinyl cation assumes different conformations in the active sites of Ms(−)-LimS and BPPS. The interconversion of the equatorial and axial α-terpinyl cations also shows an opposite preference in Ms(−)-LimS and BPPS (Figure 4).

Decomposition of the binding energies reveals, in addition to predominating electrostatic effects, a significant van der Waals component. In other words, the whole active site architecture in these enzymes is constructed to enforce a particular conformation of the α-terpinyl intermediate, thereby dictating the identity of the final product.

**Figure 5:**
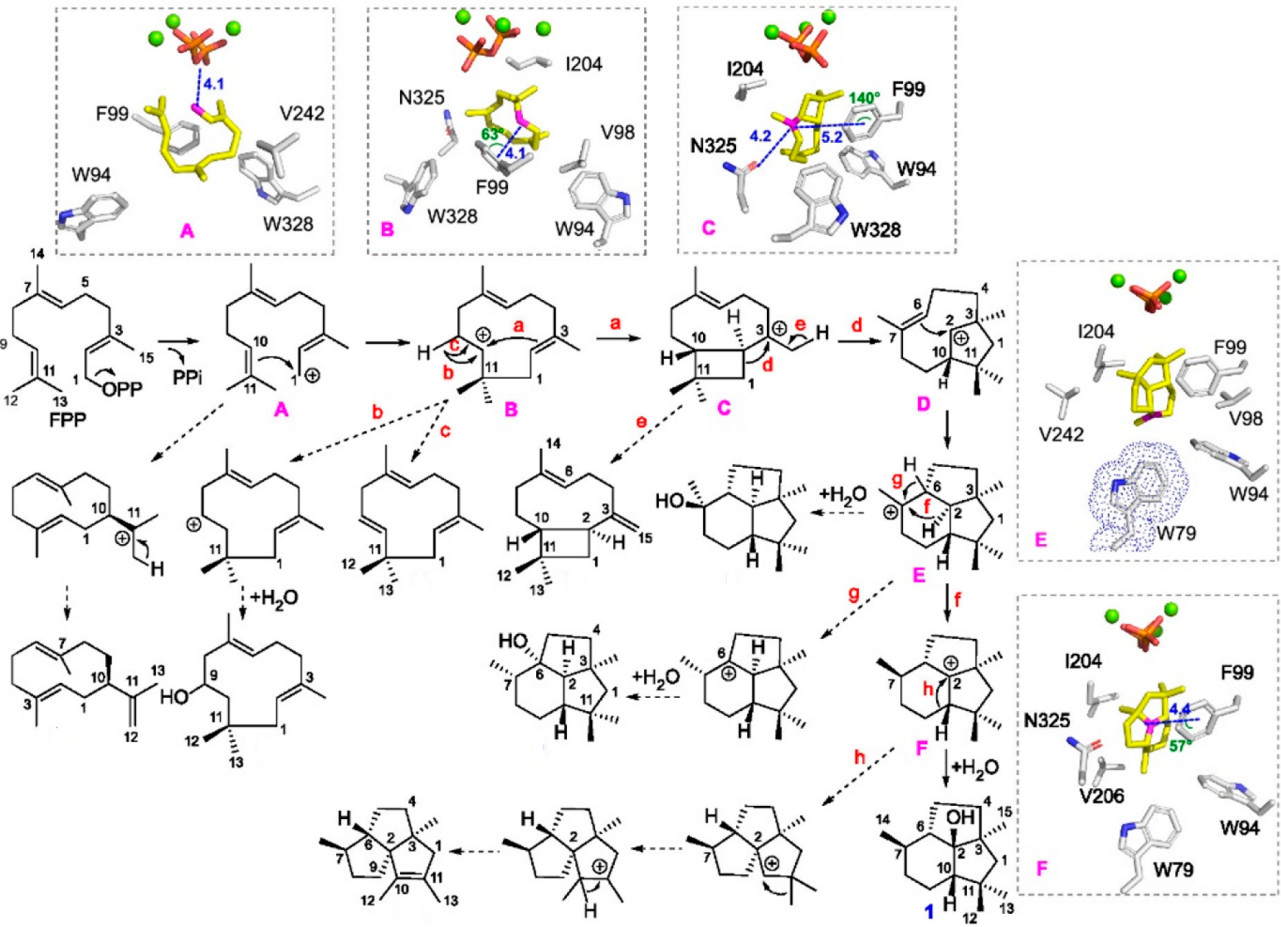
Biosynthesis of presilphiperfolan-8β-ol (1) by BcBOT2. The solid arrows show the main reaction pathway with side products (from variants) reached by dashed arrows. Red letters (a–h) represent the different reaction pathways. QM/MM MD simulations of BcBOT2 and the carbocationic intermediates (A–F, pink) are shown in the dashed boxes (cation D is a transition state with a very short lifetime). Reprinted and modified with permission from ref (116). Copyright 2023 American Chemical Society.

Many relevant terpenoids contain polycyclic carbon scaffolds. Until recently only five structures had been solved for sTSs which produce tricyclic sesquiterpenoids,^111−115^ with more structural data clearly needed. To this end, Dickschat and colleagues^116^ recently characterized three fungal tricyclic sTSs: *Botrytis cinerea* presilphiperfolan-8β-ol synthase (BcBOT2), *Dendrothele bispora* Δ^6^-protoilludene synthase (DbPROS), and *Fusarium graminearum* longiborneol synthase (CLM1). The crystal structures of these three enzymes complexed with the benzyltriethylammonium cation (BTAC), a bisabolyl cation mimic, were used as the basis for QM/MM calculations, which highlighted important active site residues (Figure 5). Using isotope labeling in both the WT and variant enzymes, the authors fully accounted for all hydride and methyl migrations during formation of the main product and all the key side products. Using a stereoselective deuteration method, they also definitively assigned the absolute configurations of all three major products.^116^

**Scheme 4:**
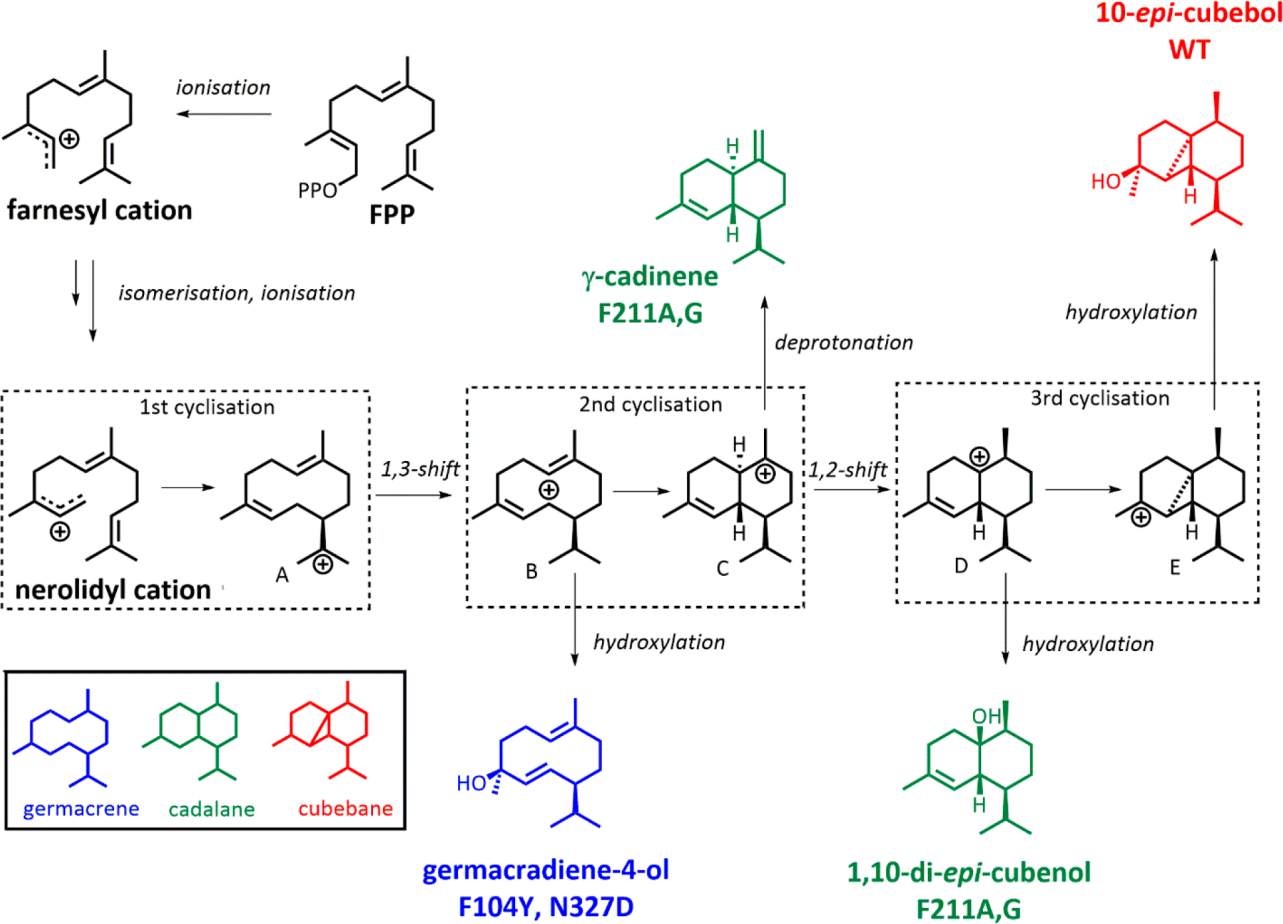
Mechanism of 10*-epi*-Cubebol Biosynthesis by ScCubS The key cyclization steps are shown in the dashed boxes. The relative distribution between cations A–E determines the carbon scaffold. Germacrenes (blue) are derived from cations A and B; cadalanes (green) from cations C and D; cubebanes (red) from cation E. Side products in ScCubS variants represent premature quenching of the reaction due to relaxed control of the intermediates. The different carbon scaffolds are shown in the bottom left box. This figure was reprinted as a modified version from ref (111) with permission under the creative commons CC-BY 4.0 license.

Elucidation of the mechanism for main and side product formation in these three enzymes showcases many of the motifs described herein. As just one example, F99 in BcBOT2 forms cation−π interactions with cations B, C, and F during the formation of presilphiperfolan-8β-ol (Figure 5). Exchange of this residue with alanine leads to significantly decreased yields of presilphiperfolan-8β-ol, and although activity was better for F99Y it was still much lower than the WT. The lack of plasticity within the aromatic family is a recurring theme in TS catalysis. This study also demonstrates the power of labeling experiments, which are attracting renewed interest due to the availability of new labeled substrates. Many of the side products produced in these kinds of studies are commercially valuable, so these results may represent a synthetic biology route to their production, but this requires an understanding of the factors that control cyclization at the molecular level. For example, we have previously speculated that an unusual serine “effector” residue in ScCubS may have coevolved with a nearby phenylalanine to allow the third cyclization step required for 10-*epi*-cubebol synthesis (Scheme 4). Exchange of this serine to the more common effector, glycine, resulted in a promiscuous enzyme that produced multiple cadalane products (bicyclic) and no cubebanes (tricyclic). This S206G variant may represent an evolutionary regression to an earlier state, before tricyclization emerged in this enzyme.^111^

Cattleyene synthase (ScCyS) is a diterpene synthase (dTS) from *Streptomyces cattleya* whose cyclization mechanism has also been studied using isotope labeling.^117^ In a further study by Dickschat and colleagues, solution of the crystal structure of apo-ScCyS and ScCyS-Mg^2+^-GGPP, the first TS crystal structure containing a natural substrate, revealed important active site residues controlling cattleyene synthesis and implied PPi as the general base that terminates the reaction. A targeted variant (C59A) was highly active but more promiscuous, producing an array of polycyclic terpenoids, one of which possessed a novel carbon skeleton. Solution of the CsCyS-C59A crystal structure revealed that in the WT the thiol of C59 pushes the phenyl ring of F86 toward the active site. In the C59A variant, the increased active site volume improves GGPP uptake but at the expense of product selectivity, by diminishing the cation−π interactions with the substrate.^118^

Since many bioactive and industrially relevant terpenoids are polycyclic, there is a need to understand cyclization in detail. The studies and results presented in this section are, therefore, fundamental to the future rational engineering of TSs.

### 2.4 Metal Cofactors, the Polar Boundary, and the Role of Pyrophosphate

In class I TSs, the Lewis acid-catalyzed abstraction of PPi by the Mg^2+^ cluster generates the reactive hydrocarbon intermediate. It was originally thought that ionization was triggered only by metal–PPi interactions, but identification of the basic residues described earlier and the so-called “effector triad” proved that this event is triggered instead by a well-coordinated interplay between the ligand and the enzyme.^93^ We will not consider the metal-binding and substrate-recognition motifs any further, as they have been described in detail elsewhere.^1^ We will, however, briefly examine the *catalytic* function of the PPi.

Isotope studies showed that in (+)-bornyl-diphosphate synthase (BPPS) from *Salvia officinalis* the C–O–P oxygen in GPP is the same as observed in the product.^119−121^ It was later shown that PPi is incorporated only into the main product and not into the side products, illustrating that it acts as part of a carefully managed molecular cascade. PPi had been proposed as a general acid/base in several TSs and is now understood to play an important role in the biosynthesis of many terpenoids.^66,122^ The active sites of TSs possess a “boundary” dividing them into two distinct environments (Figure 2). Above this boundary is the hydrophilic environment, where can be found the metal ions, metal-binding motifs, and the bound PPi part of the isoprenoid substrate. Below the boundary, the environment is hydrophobic to contain and protect the highly reactive carbocationic intermediates. The action of TSs has been characterized as an “activation-termination” process,^104^ with PPi often playing both roles.

For example, PPi is likely to act as a general acid/base in *Aspergillus terreus* aristocholene synthase (ATAS) and *Nicotiana tabacum* 5-*epi*-aristocholene synthase (TEAS), as shown using QM/MM calculations.^104^ In ATAS the PPi switches roles throughout the reaction: it acts as a general base during substrate ionization, then as a general acid during the protonation of the neutral germacrene A intermediate, and then as a general base for the deprotonation step which quenches the reaction. The carefully orchestrated role of PPi is reflected in ATAS’s higher fidelity. The lower fidelity TEAS, by contrast, has a less well-defined active site and makes use of an amino acid dyad (D444-Y520) for the same protonation.

Sometimes TSs make use of metal cofactors other than magnesium in catalysis, particularly manganese. Indeed, plant TSs such as those from conifers require Mn^2+^ as a cofactor and cannot substitute Mg^2+^ in its place,^123^ and a sTS recently characterized in the marine bacterium *Streptomyces xinghaiensis* was also found to prefer Mn^2+^, with a 3-fold increase in activity over Mg^2+^.^124^ Likewise, the concentrations of manganese were reported to be equal or greater than magnesium in Pinaceae needles,^125,126^ from which pinene, a ubiquitous and industrially relevant monoterpenoid, is obtained. This has important implications for the microbial production of terpenoids, as the optimal concentration of Mn^2+^ for mTSs has been determined at >0.5 mM, significantly higher than the cytosolic concentration in *E. coli* (∼10 μM).^123,127,128^ Interestingly, a recent study has also shown that different metal cofactors influence a functional switch between GPP and FPP synthesis in a bifunctional isoprenyl diphosphate synthase from the leaf beetle *Phaedon cochleariae*.^129,130^

Since TS activity is typically rate-limiting in high-flux conditions, there is a significant need to improve activity to prevent the accumulation of toxic intermediates. A strategy for overcoming this was demonstrated for *Pinus taeda* pinene synthase (PtPinS). Using an adapted colorimetric competition assay, a single round of mutagenesis was sufficient to isolate a PtPinS variant (Q457L) which significantly outperformed WT PtPinS in several contexts.^131^ Fascinatingly, Q457L exhibited 7× higher activity than the WT when assayed in buffer containing only magnesium due to altered geometry in the metal-binding motif. WT PtPinS uses Mn^2+^ as cofactor and therefore exhibits weak activity in *E. coli* with high cytosolic Mg^2+^ concentration. The variant, however, showed a significantly altered metal ion dependence, allowing it to function more effectively in the cytosol of the *E. coli* host. This is an important discovery with respect to the industrial production of terpenoids. Many TSs originate from plants and are expressed heterologously in microbial chassis. Although often overlooked, metal ion dependence is potentially a step-change consideration. Likewise, the other studies in this section show that the role of PPi ought not to be overlooked in any future rational engineering efforts.

### 2.5 Cation−π Interactions

TS active sites must possess a contour that is not only well-defined, capable of enforcing a particular binding mode, but also inert to protect the reactive cationic intermediates from quenching. To this end, TSs have evolved to make particular use of the large aromatic residues phenylalanine, tyrosine, and tryptophan (Figure 2). These residues define the active site contour but can also, by cation−π interactions, stabilize the positive charge on the intermediates without forming permanent bonds and prematurely terminating the reaction.^62,132−138^ Each rearrangement generates a positive charge on a different carbon atom of the substrate. Indeed, positive charge must often be directed onto a particular atom to initiate these steps in the first place, for example, to encourage attack by nucleophilic double bonds or water molecules. The electron-rich (but uncharged), aromatic π-systems of these residues can donate electron density into the empty 2p orbital of a positively charged carbon atom, permitting or encouraging the formation of charge at particular positions within the substrate. These interactions are also often observed between the aromatic π-systems and C–H bonds adjacent to positively charged carbon atoms. Consequently, a loss of aromaticity at key positions in variant TSs typically results in a reduced or even total loss of activity. In some cases, activity is preserved but a different product profile is observed, illustrating the importance of cation−π interactions for determining key branch points in the catalytic mechanism.

Alanine scanning of Ms(−)-LimS revealed that the aromatic rings of W324 and H579 are important to its catalysis, most likely by stabilizing positive charge on C7 of GPP after cyclization (Figure 1). W324 is conserved across the angiosperm lineage in TSs which make cyclic products, and H579 is conserved aromatically (H, F, or Y).^139^ W324 variants, including aromatic W324F/Y variants, produced mostly myrcene and linalool, an acyclic hydrocarbon, and monoterpene alcohol, respectively, which both represent a failure to form the cyclic α-terpinyl intermediate (Figure 1). The lack of plasticity within the aromatic family is again notable.

Similarly, in *Paeonia lactiflora* pinene synthase (PlPinS) exchange of a single phenylalanine was sufficient to convert it into a sabinene synthase. WT PlPinS makes α-pinene with 98% selectivity and <1% sabinene. F482A/V/I/T all acted as sabinene synthases, with F482A the best-performing (>90% selectivity and ∼50% activity). F482T suggests the aromatic quadrupole, and not a dipole, is required at this position. However, once again F482W/Y variants could not rescue WT activity.^140^ The carefully sculpted active site does not allow for the conformational rearrangements required to accommodate the hydroxyl or indole ring. These data suggest F482 encourages the formation of the pinyl cation, likely by stabilizing positive charge at C3 after cyclization. In the absence of this stabilization, the 6,7 hydride shift generates the terpinen-4-yl cation, which is subsequently attacked to yield the thujyl cation, deprotonation of which gives sabinene (Figure 1).

Another bicyclic monoterpenoid, (+)-3-carene, is a major constituent of defensive oleoresin, and is believed to provide resistance to the white pine weevil, a major pest of North American pine and spruce.^141^ Three related (+)-carene synthases (PsCarS) and a related sabinene synthase (PsSabS) from *P. sitchensis* can essentially be interconverted by mutation of a single residue. Reciprocal mutation of L596F in PsCarS conferred PsSabS-like product profiles and vice versa.^22^ Consisting of fused cyclopropane and cyclohexene rings, (+)-3-carene is achieved from the deprotonation and subsequent 5,7 intramolecular cyclization of the key α-terpinyl cationic intermediate (Figure 1). By contrast, the related bicyclic monoterpenoid (−)-sabinene proceeds through the aforementioned thujyl cation. In PsSabS, F596 stabilizes the formation of the terpinen-4-yl cation required for (−)-sabinene synthesis by cation−π interactions. This was demonstrated by an F596H variant with WT-like product profiles. An F596E variant made mostly limonene, consistent with deprotonation by the negatively charged glutamic acid residue at C8, while an F596G variant gave a range of products, consistent with relaxed steric and electronic control of the substrate. By contrast, in PsCarS the leucine at position 596 is not capable of providing electronic control; after deprotonation at C5, intramolecular cyclization yields (+)-3-carene. In both PsSabS and PsCarS relatively high amounts of terpinolene are observed, indicating that neither enzyme has evolved exact control of the deprotonation step at this part of the reaction. This could be deliberate, however, since defensive oleoresins typically consist of dozens of different terpenoid compounds.^22^

When the length of the substrate increases, so does the potential complexity of the reaction. ScCubS converts FPP to the tricyclic sesquiterpenoid 10-*epi*-cubebol at >90% purity, indicating a high level of catalytic control. The proposed mechanism of 10-*epi*-cubebol formation (Scheme 4) illustrates how manipulating the position of the positive charge is fundamental to preventing quenching and achieving the final product.^111^ F104 was found to be important for stabilizing the formation of cation C after the second cyclization. Exchange of this residue, even with other aromatic residues, resulted in premature reaction termination. The disrupted active site in the F104Y variant was attributed to changes in the local hydrogen-bonding network due the presence of the new phenolic hydroxyl group, consistent with other results.^142^ There is a kinetic distribution between the various cations along the reaction coordinate, which is driven forward by multiple TS motifs, most importantly cation−π stabilization of key intermediates. Aromatic residues with a presumed more steric role, for example, F211, can to some extent tolerate being mutated to aliphatic residues like leucine, although aromatic substitutions were much more WT-like. F104, however, cannot tolerate these changes, even to the other aromatic residues, consistent with the conservation of particular aromatic residues in analogous TSs.^143^

**Figure 6:**
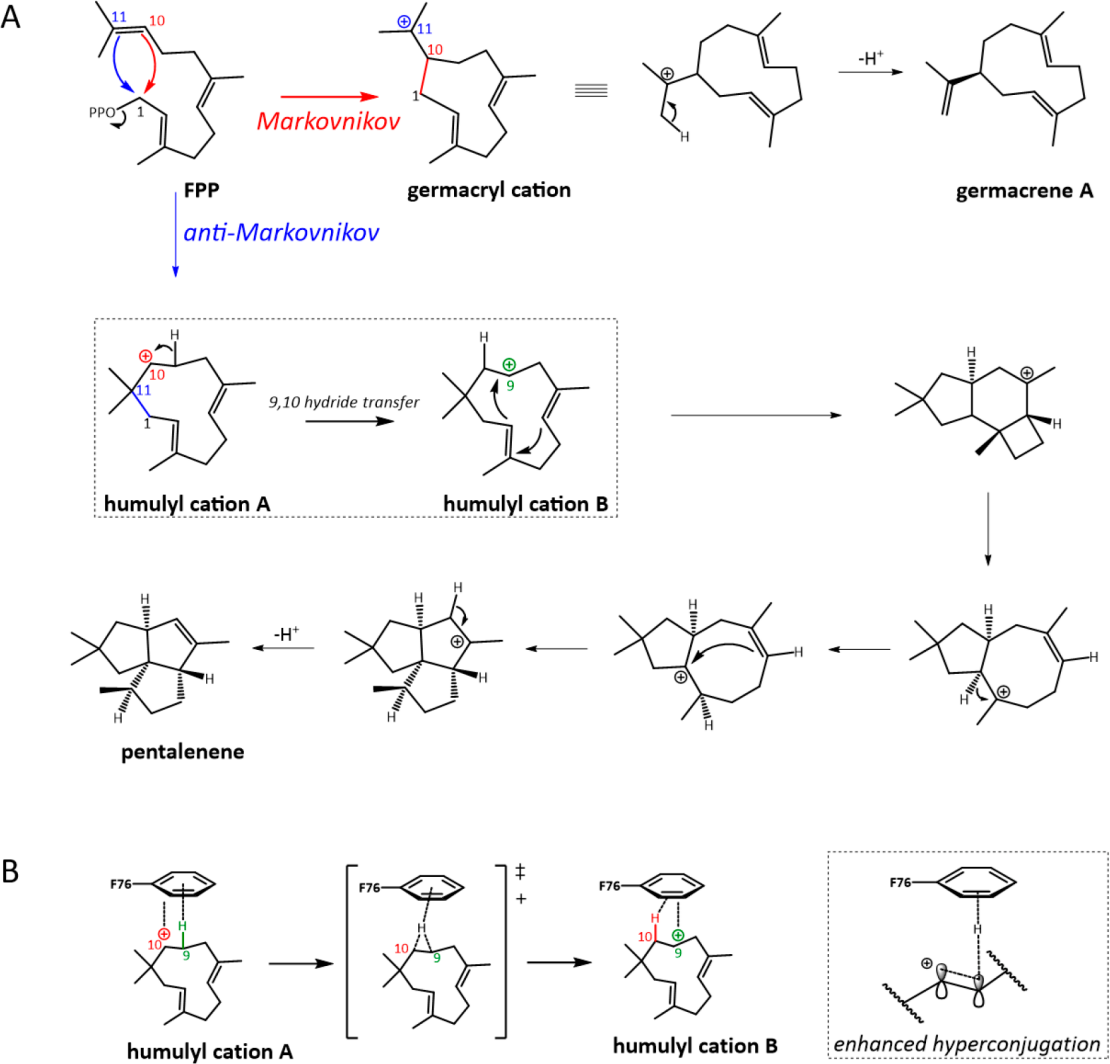
(Anti-)Markovnikov addition in TSs. (A) Most TSs proceed via Markovnikov addition (red pathway), for example, 1,10 ring closure of FPP to generate a tertiary carbocation on C11 during germacrene A biosynthesis. Pentalanene synthase, however, proceeds via anti-Markovnikov (blue pathway) 1,11 ring closure to generate a secondary carbocation on C10. The key 9,10 hydride transfer in pentalenene synthesis is shown in the dashed box. (B) Enhanced hyperconjugation from the C9–H–F76π interaction stabilizes the transfer of charge from C10 to C9 after anti-Markovnikov addition.

Cation−π interactions are also fundamental to TSs that proceed via anti-Markovnikov addition. When the TS substrate cyclizes via attack of the positive charge by a double bond, Markovnikov’s Rule states that the resulting positive charge should be on the more substituted carbon, stabilized by a greater degree of induction and hyperconjugation from neighboring carbon atoms. However, some TSs preferentially proceed via less-favored anti-Markovnikov intermediates due to the architecture of the active site and the positioning of key aromatic residues. The model sTS PS produces an enantiomerically pure triquinane with no fewer than four stereogenic centers and catalyzes the first committed step in the formation of pentalenolactone antibiotics in several *Streptomyces* species. Notably, experiment and quantum calculations have shown that PS undergoes an initial 1,11 anti-Markovnikov cyclization to yield the secondary humulyl cation, in favor of 1,10 Markovnikov cyclization which would yield the tertiary germacryl cation, contrary to what occurs in most TSs and what would be expected in solution (Figure 6).^144^ Crystals of PS with a fluorinated analogue allowed Oprian and colleagues to uncover a mechanism for anti-Markovnikov cyclization which depends on cation−π stabilization.^103^ C9 of FPP is perfectly positioned to make a 2.5 Å C–H−π interaction with the aromatic ring of F76 (Figure 6), which enhances hyperconjugation and stabilizes the development of positive charge on the neighboring C10 (the consequence of anti-Markovnikov cyclization). This aromatic residue then promotes migration of a hydride from C9 to C10, the second step in pentalenene synthesis.

An F76W variant makes a significant amount of α-humulene, a second type of anti-Markovnikov product resulting from deprotonation at C9 (Figure 6). The crystal structure of this variant-analogue complex shows that C9 and C10 of FPP are centered above the benzene and indole rings, respectively, meaning F76W can stabilize both, hence the two anti-Markovnikov products. Intriguingly, it could also be that the indole ring directly participates in the deprotonation to form α-humulene. The authors note that there is a rough correlation between *Streptomyces* TSs possessing an aromatic residue at position 76 and making anti-Markovnikov products. This approximate correlation with multiple exceptions is typical of TSs, again illustrating the difficulty of rational engineering from sequence alone.

**Scheme 5:**
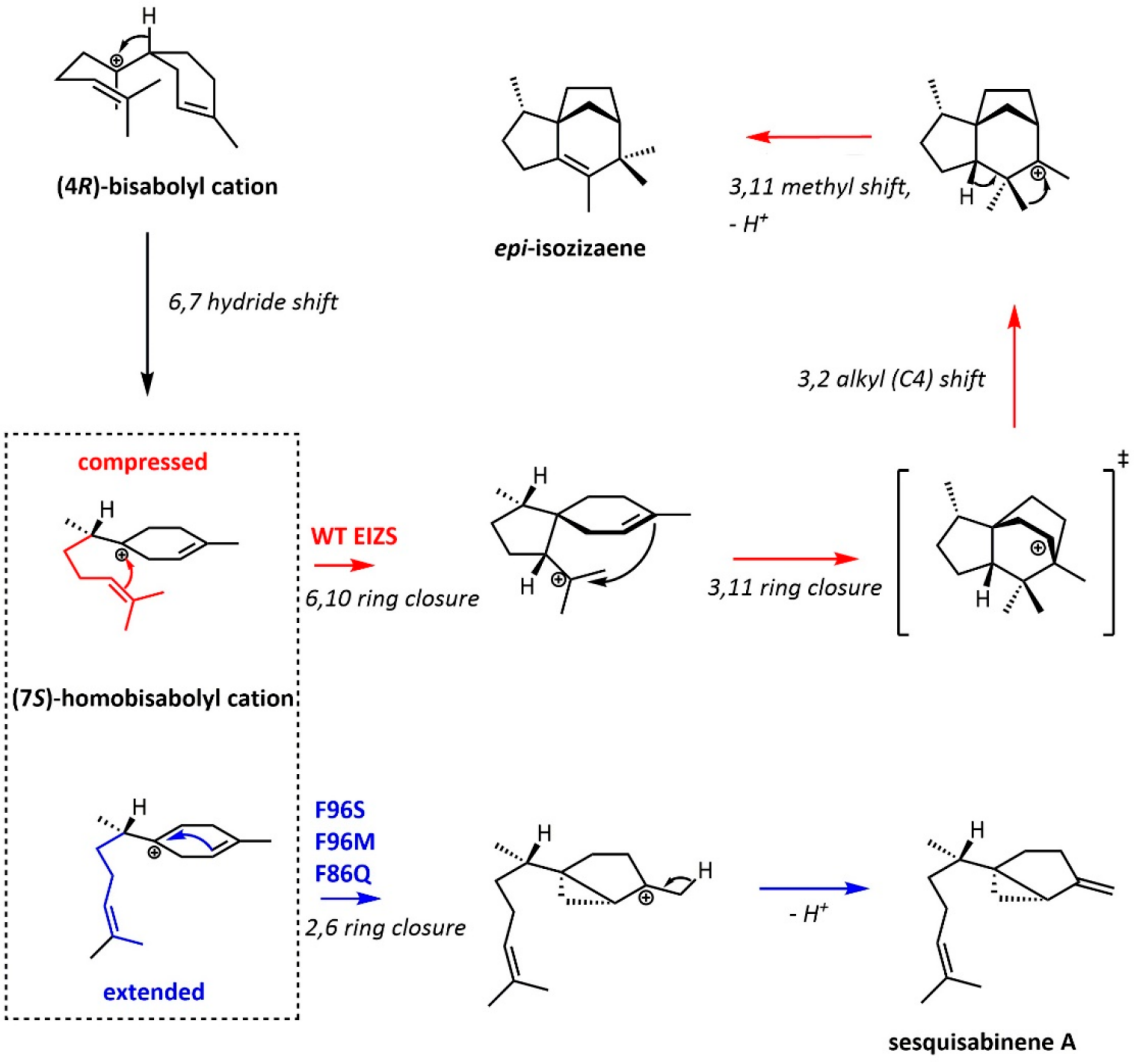
Mechanisms for WT EIZS and F96 Variants The (7*S*)-homobisabolyl cation is a branching point. Spirocyclization of the compressed (red) conformation eventually leads to *epi*-isozizaene, whereas the extended conformation (blue) yields a strained bicyclic cation which is deprotonated to give sesquisabinene A.

Aromatic residues are often found in TSs as part of subtle networks, the perturbation of which can have dramatic effects on catalysis. The sTS *epi*-isozizaene synthase (EIZS) from *S. coelicolor* catalyzes the conversion of FPP to *epi*-isozizaene, the tricyclic precursor of the antibiotic albaflavenone.^112,145,146^ Previous work by Christianson and colleagues has shown that EIZS variants F96S/M/Q all become high-fidelity sesquisabinene A synthases.^147^ In a further study, they crystallized WT EIZS, F96M, and F96S with a combination of BTAC, Mg^2+^ ions, PPi, and diphosphate-containing inhibitors, catching different open and closed states which result in different active site conformations and volumes.^148^ Although overall these active sites are quite similar, the production of *epi*-isozizaene vs sesquisabinene A is determined at a key branching point with the (4*R*)-bisabolyl cation, which can be perturbed with only small changes to the active site (Scheme 5).

F96 is part of an aromatic triad (F95, F96, and F198) which largely defines the active site contour and is part of an extended aromatic cluster.^112,149^ The residues in this cluster define the active site or interact with residues that do so. In the F96M variant, a loosened active site means that the isoprenoid tail of the (4*S*)-homobisabolyl cation cannot be held sufficiently close to the positive charge, yielding an alternative bicyclic cation, deprotonation of which yields sesquisabinene A (Scheme 5). F96S substitution weakens the cation−π interactions between the substrate analogue amino group and the two other aromatic residues of the triad, F95 and F198. This change in substrate orientation is cascaded through the cluster, affecting the cation−π interactions between it and the substrate. The authors therefore characterize F96 as an electrostatic toggle: it has a steric and electronic role both in closing the active site and defining the active site contour as part of an extended cluster. These interactions are perturbed by F96 mutation, and although F96M can to some extent make up for this with S−π interactions, it cannot compensate for the loss of π-stacking interactions.

**Scheme 6:**
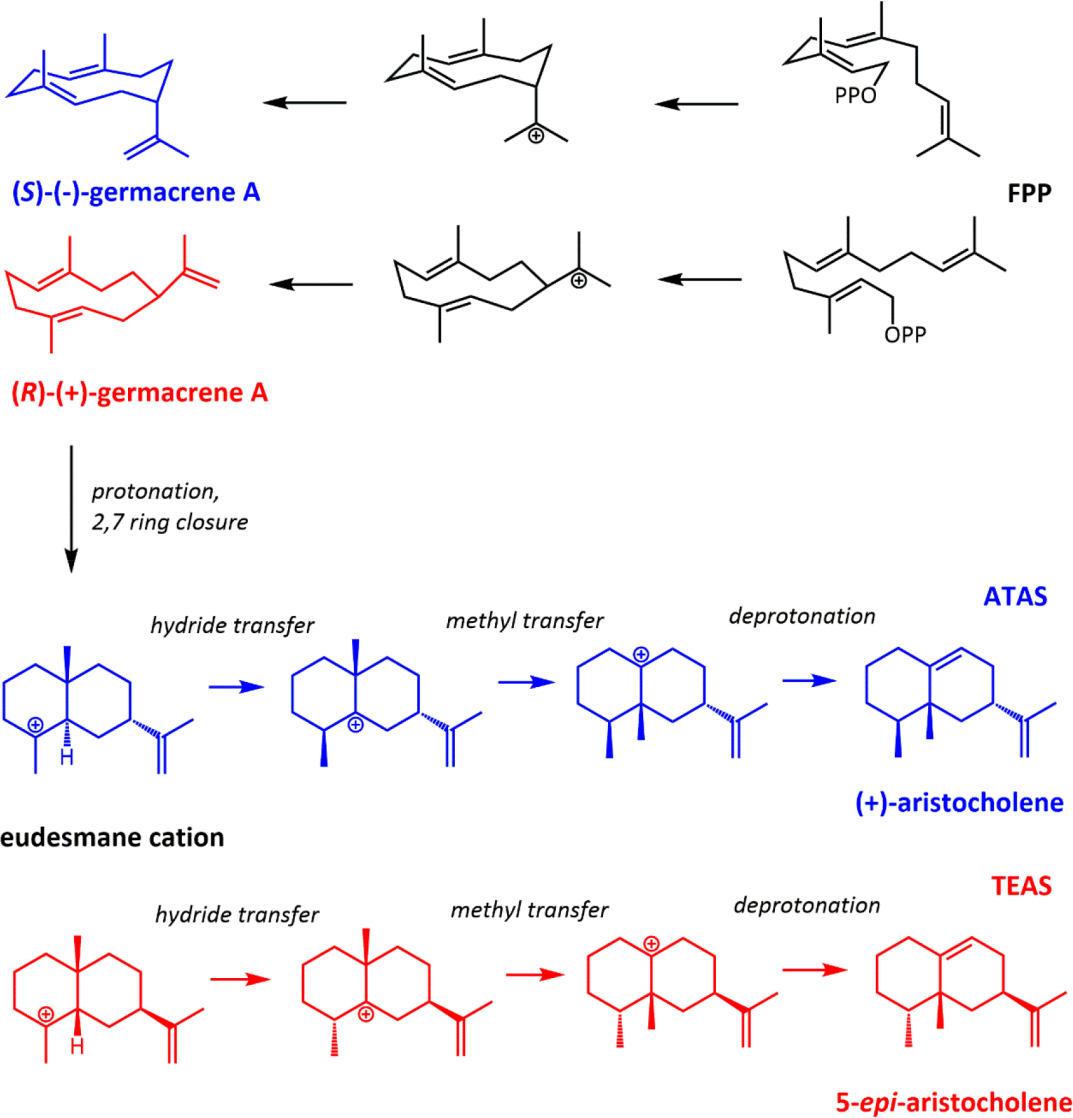
Catalytic Mechanisms of ATAS and TEAS ATAS (blue) and TEAS (red) proceed through different enantiomers of the neutral germacrene A intermediate, which ultimately yields different enantiomers of aristocholene.

The correlation between TS fidelity and the composition of the active site is an important one, not least for the rational design of TSs. Enzyme fidelity in TSs owes both to steric control and electronic interactions, with different aromatic active residues playing different roles.^150^ ATAS and TEAS produce different stereoisomers of the sesquiterpenoid aristocholene from FPP (Scheme 6). Both proceed via 1,10 intramolecular cyclization to yield the germacryl cation, and subsequently the neutral intermediate germacrene A. However, ATAS proceeds through (*S*)-(−)-germacrene A, and TEAS through (*R*)-(+)-germacrene A. Reprotonation, a series of hydride and methyl transfers, and a final deprotonation step yields either (+)-aristocholene (ATAS) or 5-*epi*-aristocholene (TEAS), depending on the stereochemistry of the germacrene intermediate.^104^

ATAS is high-fidelity, and TEAS is promiscuous. The active site of ATAS is enriched with aromatic residues, whereas TEAS contains unusual aromatic residues, at least compared to analogous structures.^151,152^ This likely also explains TEAS’s ability to process the *cis-*form of FPP, a non-native conformation for the substrate.^153^ Zhang and colleagues have shown that F81 and F147 contribute to fidelity in ATAS by more strictly defining the active site and making cation−π interactions with the evolving substrate.^104^ The protonation (and subsequent cyclization) of the neutral germacrene A intermediate to yield the eudesmane cation (Scheme 6) has essentially the same energy barrier in ATAS and TEAS: 15.3 and 15.5 kcal/mol, respectively. However, in ATAS 5 kcal/mol more heat energy is released upon achieving the eudesmane product as a result of strong cation−π interactions between the eudesmane cation and F81, with F147 maintaining the substrate in an optimally reactive binding pose throughout the reaction by steric hindrance. The analogous Y520 in TEAS, by contrast, does not provide π-stacking interactions with the germacrene A intermediate. The eudesmane cation then undergoes intramolecular 1,2 hydride shift and then methyl transfer to yield the precursor to the final (enantiomeric) aristocholene products. In TEAS, these steps have energy barriers of 7.8 and 3.8 kcal/mol, respectively, and formation of the aristocholene precursor is exergonic by 4.2 kcal/mol with respect to the eudesmane cation. In ATAS, the same two steps are much more facile, with energy barriers of 3.0 and 0.9 kcal/mol, respectively, and formation of the precursor is exergonic by 7.6 kcal/mol with respect to the eudesmane cation, indicating kinetic and thermodynamic favorability. The authors also note that their computed orientation for the final intermediate in ATAS is very similar to an ATAS–analogue complex reported by Christianson.^152^ Consistent with our remarks in section 2.2, the authors note that the carbocation migrations are feasible in the first place due to the high intrinsic reactivity of the carbocation itself but that the extrinsic enzymatic environment plays a crucial guiding role.

It is notable that TEAS has higher enzyme activity than ATAS, again implying that TSs make a trade-off between fidelity and activity, a typical example of “negative catalysis”.^79^ The ability of the aromatic residues to restrict substrate flexibility and stabilize key intermediates likely explains their ubiquity and conservation in TSs. Indeed, the authors highlight a rough correlation between the number of aromatic residues in the active site of a TS and its fidelity. They also note that fungal and bacterial TSs tend to have more active site aromatic residues and likewise tend to be higher fidelity. There are exceptions to this, however, and the authors concluded that only aromatic residues with essential catalytic roles ought really to be considered in relation to fidelity.

### 2.6 Water Addition, General Catalysis, and the Importance of Polar Residues

The defining feature of TSs is that they handle highly reactive carbocationic intermediates inside a hydrophobic active site. However, polar residues have been shown to play an important role in TS catalysis. An obvious role for a polar residue in TSs is to quench the reaction by deprotonating the final intermediate. This is the same role played by PPi in many TSs. However, some TSs incorporate a water molecule, and some TS mechanisms have now been shown to proceed via a neutral intermediate which requires protonation at a key point in the reaction.^104^ TSs have clearly evolved a careful strategy for guaranteeing the product outcome, making use of key polar amino acids embedded in a mostly hydrophobic active site. Mutation of these polar residues therefore often results in altered product profiles. For example, in Ms(−)-LimS Y573 was found to be crucial for enzyme activity via both its aromatic ring *and* its hydroxyl group: Y573V was inactive, and Y573F showed only 2.79% of WT activity.^154^ Likewise, Y573D and Y573S were both inactive. A key hydrogen bond between Y573 and D496 was found to play a role in achieving the correct substrate confirmation, as discussed in section 2.3.

Christianson and colleagues have previously shown that substitution of nonpolar for nonpolar residues remolds the active site of EIZS and enhances chemodiversity.^149^ In a later study, they swapped nonpolar residues for polar ones to make a series of new variants. These generate various product arrays, and some of them produce hydroxylated products (no hydroxylated products are observed in WT EIZS). Three of the variants (F96Q/M/S) become high-fidelity sesquisabinene A synthases, as discussed above, also demonstrating that the addition of polarity does not automatically result in the formation of polar products. These polar moieties can be rendered totally inert by being placed outside of a reactive conformation with respect to the substrate, highlighting that rational incorporation of polar residues into TSs as a means of modulating product outcome might be a more useful tool than previously imagined.^147^

**Scheme 7:**
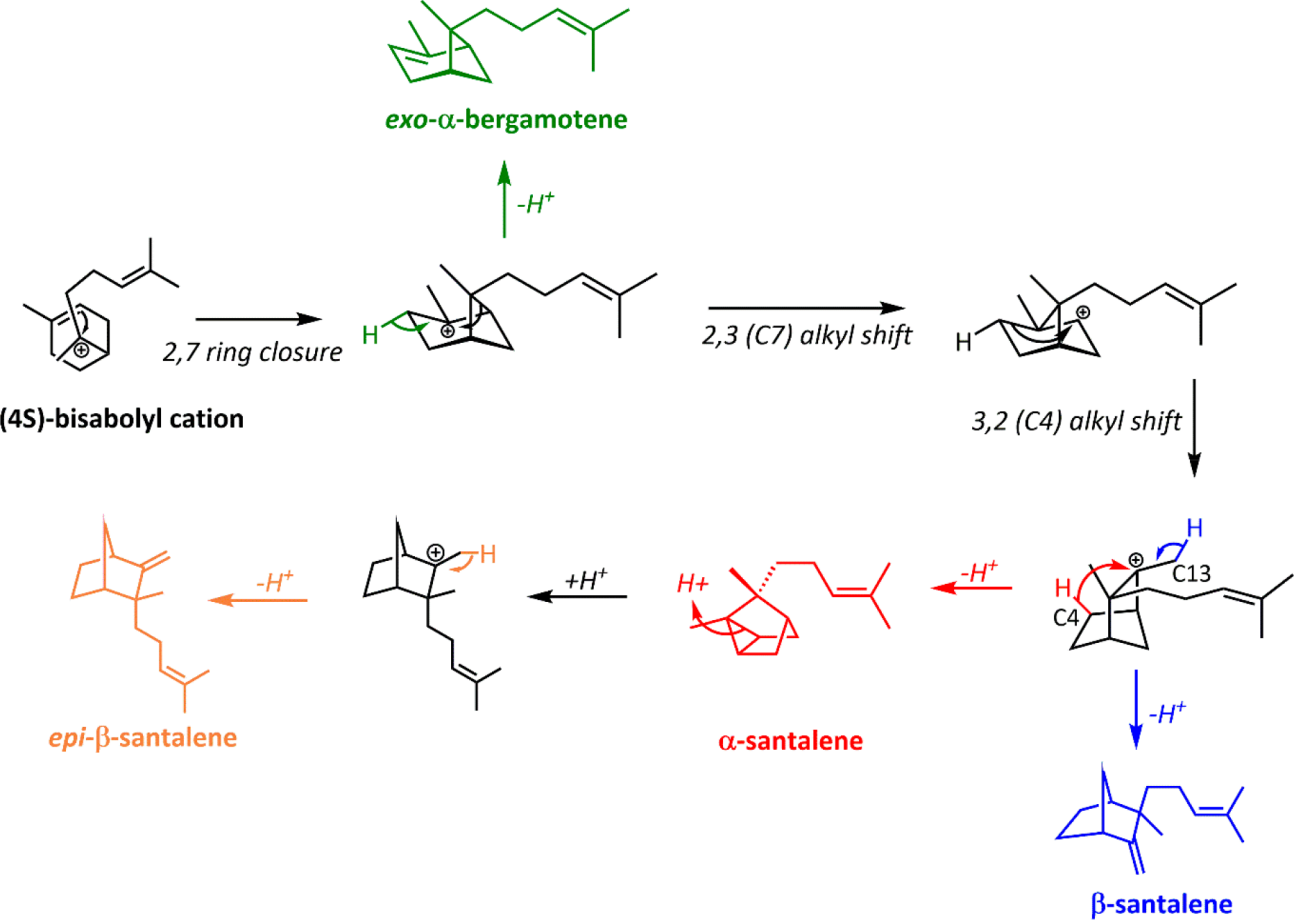
Santalene Biosynthesis by SanSyn and SaSSy SanSyn is high-fidelity and more tightly controls the (C4) deprotonation step leading to α-santalene (red). SaSSy promiscuously deprotonates at C4 and C13 (blue).

In fact, polar residues help control everything from active site shape and size, water incorporation, (de)protonation, substrate binding, and even whether a TS is high-fidelity or promiscuous. They can also stabilize carbocations via cation–dipole interactions. SanSyn from *Clausena lansium* and SaSSy from *S. album* are a high-fidelity and a promiscuous santalene synthase, respectively. SaSSy converts FPP into a mixture of α-santalene, β-santalene, *epi*-β-santalene, and exo-α-bergamotene at an approximate ratio of 4:2:1:3, where SanSyn is essentially a pure α-santalene synthase (Scheme 7). QM/MM and mutagenesis reveal that in both enzymes a threonine residue (T298/T318 SanSyn/SaSSy) acts as a general base during the key deprotonation step which yields α- or β-santalene.^155^ T298/318 are conserved in both enzymes despite only 33% sequence identity, and T298/T318 alanine variants lost the ability to make santalenes. However, proper deprotonation of the cationic intermediates to yield santalenes depends on more than just the presence of this catalytic base. In SanSyn, F441 plays a key role in restricting the conformational freedom of the important bisabolyl intermediate, reducing the opportunity for side product formation. Additionally, the isoprenoid tail of FPP is correctly oriented to benefit from stabilizing hyperconjugation. This strictly enforced orientation results in a more controlled deprotonation, with T298 predominantly abstracting a proton from C4 (α-santalene) instead of from C13 (β-santalene). In SaSSy, the corresponding S459 allows greater substrate freedom, meaning that deprotonation by T318 can occur at more than one position. On this basis the authors engineered a promiscuous and catalytically efficient SanSyn variant (F441V) which produced 57.2% α-santalene and 28.6% β-santalene, but only 6.7% *epi*-β-santalene and 7.6% *exo-α*-bergamotene, a desirable ratio for the commercial production of *S. album* essential oils.^155^

This subtle interplay is more typical of TSs than a polar residue acting as a simple acid or base. The relationship between active site volume and the incorporation of water in particular has emerged as an important one. For example, δ-cadinene synthase from *Gossypium arboretum* (GaDCS) is a high-fidelity sesquiterpene synthase which achieves 98% δ-cadinene from FPP with 2% formation of the germacrene alcohol germacradien-4-ol (Gd4ol) as a side product. A previous study by Keasling and colleagues had shown that a double mutant (N403P/L405H) turned GaDCS into Gd4ol synthase (93% purity), but with significantly impaired catalytic efficiency.^156^ In a more recent study, Alleman and colleagues discovered a single residue mutation (W279A) capable of turning GaDCS into a catalytically efficient Gd4ol synthase (>80% Gd4ol, 11% δ-cadinene).^157^ W279 and the nearby Y410 are located closer to the substrate than the previously identified N403/L405 pair and are ideally placed to form hydrophobic interactions that may control the binding conformation of FPP. Gd4ol synthesis depends on water attack on the germacryl cation, but for δ-cadinene synthesis this must be blocked to allow intramolecular cyclization and subsequent deprotonation. W279 presumably blocks the water attack that would quench the reaction (and may promote deprotonation as a catalytic base). Although W279 may help stabilize the germacryl cation by cation−π interactions, variants at this position revealed a near-linear correlation between the van der Waals volume of the residue and the proportion of hydroxylated products. This is consistent with the presumed role of W279 in preventing hydroxylation by steric blocking. Polar variants at W279 affected product ratios (according to size) but not catalytic efficiency, further suggesting the size of residue 279 defines a water channel to the active site but otherwise does not play an important role in determining the reaction products.

When searching for candidate general acids and bases, the natural place to start is with polar residues. However, the recent literature continues to imply the existence of an atypical base in TS catalysis, namely, tryptophan. It was noted above that W279 appeared to promote deprotonation in GaDCS, although this effect is difficult to separate from its role in defining the water channel to the active site. In other studies, however, this role for tryptophan has been suggested more convincingly. For example, in Ms(−)-LimS, Srividya and co-workers ruled out PPi as the general base for deprotonation because it is too far away (in contrast, for example, to BPPS^119^). H579 has the properties of a catalytic base and is positioned suitably for deprotonation, but mutagenesis revealed it can be exchanged with nonbasic residues with only a moderate impact on activity. W324 variants, by contrast, are substantially worse.^139^ It is worth noting that the α-terpinyl intermediate is a strong conjugate acid with p*K*_a_ = −10.

Similarly, in ADS, W271 is implicated as the active site base.^106^ There is a loss of activity in all ADS W271 variants, even aromatic ones. Furthermore, an identical result was reported for the analogous W273 in TEAS.^10^ Positive charge on C11 of the amorphenyl cation increases the acidity of the proton at C12 pointing at W271, which would again become a positively charged arenium ion (WH^+^), presumably driven by the favorability of quenching the highly reactive carbocationic intermediate. The hydroxyl group of T399 was also shown to be important, with W271 and T399 working in tandem in ADS to ensure amorpha-4,11-diene synthesis, a partnership observed earlier in the santalene synthases SanSyn and SaSSy.

One residue with unambiguous significance in TS catalysis is asparagine. This moderately polar residue appears time and again in the active site of TSs, and its importance has been demonstrated in multiple studies: it can stabilize the intermediates by cation–dipole interactions and bind catalytic water molecules without being at risk of permanent alkylation. However, as will be discussed, its position is not necessarily conserved. It is also worth noting that this residue is separate from the asparagine of the NSE triad, although the asparagine from this metal-binding motif can also play a role in the reaction beyond coordination of a metal ion, such as in bCinS where N220 of the NSE triad helps coordinate a relevant water molecule.^57^

In bCinS, mutagenesis, MD simulations, and QM/MM calculations showed that N305 is crucial for 1,8-cineole formation.^57^ The analogous N338 in SfCinS was previously found to be essential for forming the key α-terpinyl intermediate.^19^ N305 in bCinS is located on the opposite side of the active site relative to N338 in SfCinS, reflected in the opposite stereochemical pathways pursued by each enzyme, as discussed in section 2.1. bCinS N305 variants are either inactive or make nonhydroxylated/noncyclized products, and MD simulations show that the water molecule coordinated by N305 in the bCinS crystal structure is only retained in the WT. Similarly, with respect to α-terpinyl formation, the distance between the relevant C7 of GPP and the nearest water molecule shows a strong peak at 4 Å for the WT, whereas this distance broadens for the variants, and in some cases the water molecule is not coordinated at all.

In Ms(−)-LimS, a single N345A mutation was sufficient to turn the enzyme into a pinene synthase. The polarity of N345 was once again concluded to stabilize the α-terpinyl intermediate, allowing deprotonation which leads to limonene (Figure 1). PPi could stabilize the pinyl cation if cyclization were to outcompete deprotonation; it is therefore proposed that removing polarity at position 345 nudges the reaction in this alternative direction. Xu and co-workers propose a “polar pocket” in which N345 is a (main) contributor to stabilizing the α-terpinyl intermediate, as alanine variants of the other residues in this pocket also resulted in less limonene and/or more pinene. They also note that in many limonene synthases the following motif is preserved: when position 345 (equivalent) is asparagine, 321 is always a cysteine, but if 345 is isoleucine or cysteine, 321 is always serine or tyrosine. In other words, it seems that if polarity is absent at position 345 it must be compensated for at position 321.^23^

The importance of asparagine can also be proven by the opposite result: namely, that *increased* polarity at this position also derails the reaction. In ScCubS, N327 is crucial for catalysis by promoting the first of three cyclizations required to reach the cubebane carbon scaffold (Scheme 4). An N305A variant destroyed enzyme activity, as observed for bCinS.^19,57^ However, a substantially altered product profile was also observed for the N327D variant in which a relatively large amount of germacradien-4-ol (Gd4ol) was accumulated. Gd4ol results from the premature nucleophilic quenching by water of the first cyclic intermediate, as in GaDCS.^157^ The explicit negative charge on N327D promotes this reaction by altering the kinetic distribution between the early mono- and bicyclic intermediates, stalling the reaction, and permitting the formation of side products. N327 also binds a water molecule in the ScCubS crystal structure, suggesting that N327D may polarize this water to a greater extent, further encouraging the quenching reaction (Gd4ol is hydroxylated at the same position as 10-*epi*-cubebol). As such, the choice of asparagine in ScCubS is highly specific: it provides sufficient stabilization to enable the first cyclization, but its moderate polarity does not prevent the further steps required for 10-*epi*-cubebol synthesis.^111^

**Figure 7:**
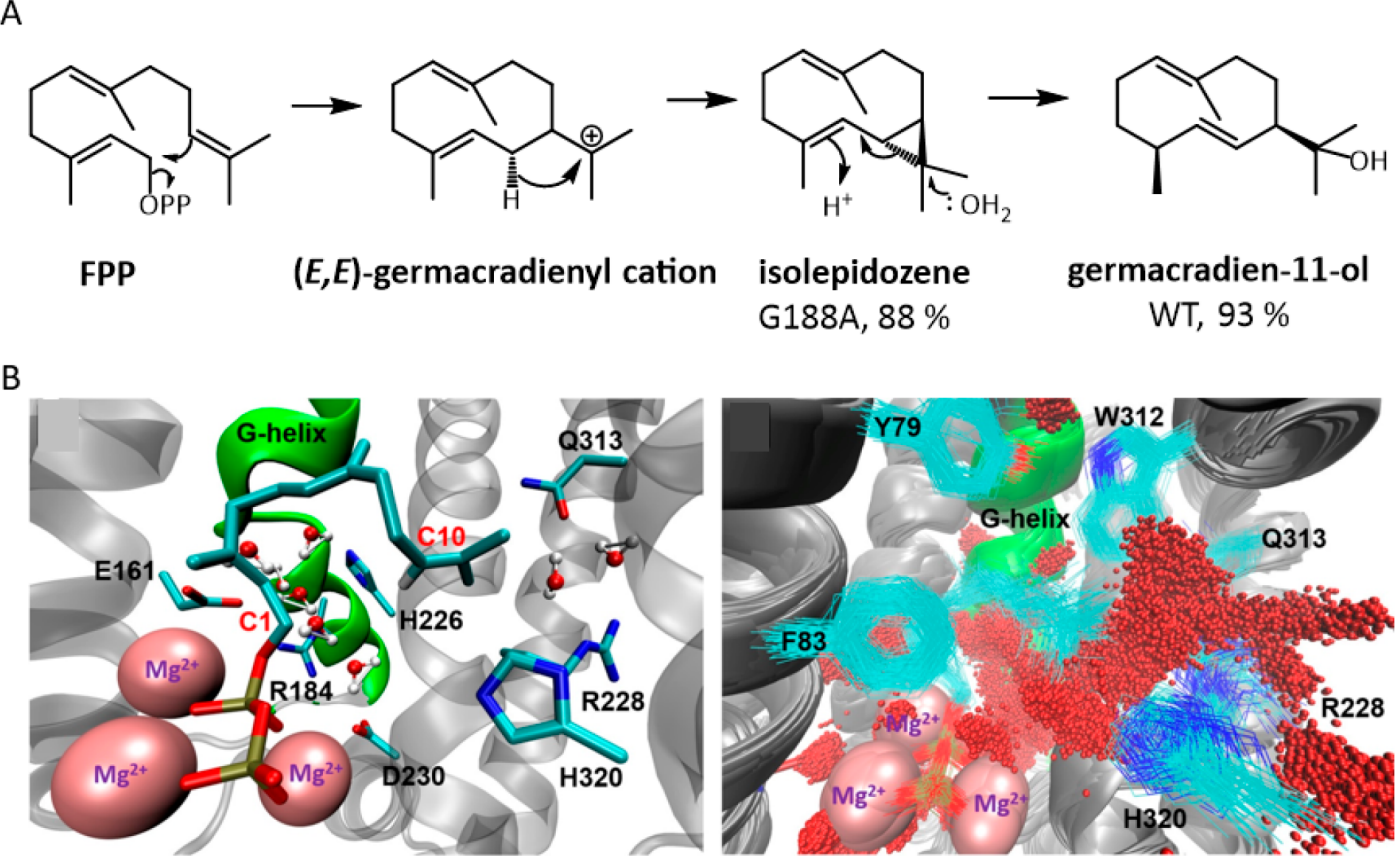
Water capture in Gdol11S. (A) Reaction mechanism for germacradien-11-ol biosynthesis by Gdol11S. The reaction proceeds through the neutral intermediate isolepidozene and can be terminated here with changes to the active site water channel. (B) The two major water sites in the active site of Gdol11S (RQH and G-helix). Left: representative MD snapshot showing water molecules at the two sites. FPP (teal) is shown bound to the Mg^2+^ cluster (pink). Right: superimposition of the enzyme–substrate complex during one of the MD simulations at 4 (water) and 200 ps (system) intervals. The red spheres represent the location of water molecules across the simulation, which flow between the two sites. Panels B and C were reprinted without modifications from ref (158) with permission under the creative commons CC-BY 4.0 license.

We have seen that water molecules can enter the active site and are yet not incorporated into the final product. Conversely, sometimes water is incorporated without any clear candidate for doing so. The picture is further complicated by the discovery that water management can depend on residues and structural motifs located far from the substrate. An exemplary case is that of the sTS germacradien-11-ol synthase (Gd11olS) from *S. coelicolor*, which van der Kamp and colleagues have shown proceeds via the neutral intermediate isolepidozene (Figure 7).^158^ MD simulations revealed W312 and H320 (note again tryptophan and a polar residue) likely played a role in forcing the precyclic conformation: residue exchanges at these positions led to an increased amount of acyclic nerolidol. However, H320A did not dramatically alter the product profile, indicating that this is likely not the base required to deprotonate the germacryl cation to the neutral isolepidozene. The prochiral selectivity observed in WT simulations is disrupted in alanine variants, owing to a loosened active site pocket and relaxed control of the substrate. Intriguingly, W312F permits the cyclization of FPP to the germacryl cation, but not the conversion of this cation to isolepidozene, once again implicating an active site tryptophan as a general base, although the authors do not pursue this. They do reveal, however, that water is found in two major regions close to FPP during simulation: the “RQH” site (R228, Q313, and H320) adjacent to C11 of FPP, and the G-helix. Water flows freely between these regions during the simulations, but although water exchange is observed between the G-helix and bulk water, a similar exchange is not observed between RQH and bulk water. In other words, it appears that water enters the active site via the catalytically important G-helix and then can move to the RQH site (Figure 7). Perturbation of the G-helix is known to have profound effects on TS catalysis, as reported for S206G variants of 10-*epi*-cubebol synthase.^111^ Nonpolar Gdol11S RQH variants showed increased accumulation of nonhydroxylated products, but a significant change was only observed for H320F, probably due to its increased size blocking access of water to FPP (similar to the channel described above for GaDCS). H320 is therefore the most important residue of the RQH site, but none is implicated as a general base. Mutation of the G-helix was much more dramatic. When G188 (the effector in Gdol11S) was exchanged for alanine, the variant accumulated 88.4% of the neutral intermediate isolepidozene (Figure 7). The importance of the G-helix in forming the Michaelis complex in TSs is well-established and is illustrated here by the >8-fold increase in *K*_M_ for the G188A/S and A190V variants. Simulations of G188A/S show that water availability to FPP is not the explanation for altered product profiles (e.g., as for the RQH site). Rather, changes in conformation around the G-helix owing to altered hydrogen bonding networks and backbone orientations subtly affect water interactions along the reaction coordinate,^158^ consistent with results reported for selinadiene synthase.^159^ Understanding these subtle and dynamic effects will be crucial for any engineering efforts, especially for the production of bioactive terpenoids.

Finally, in this section we acknowledge a finding reported for a maize TS which is capable of directly performing double hydroxylation of FPP. Incorporation of more than one oxygen atom into terpenoids typically requires enzymatic processing downstream of TSs, for example by P450s, but a eudesmane-2,11-diol synthase from *Zea mays* (ZmEDS) directly achieves a dihydroxylated product.^160^ A phenylalanine at the base of the active site was found to be crucial for eudesmane-2,11-diol synthesis, and although its role was not definitively assigned, it is believed to position and/or activate the neutral hedycaryol intermediate for protonation, the key step to generating a new cation for the second hydroxylation step. Interestingly, when ZmEDS is fed hedycaryol as a substrate it cannot convert it to eudesmane-2,11-diol, consistent with 5-*epi*-aristocholene synthase when fed germacrene A.^161,162^ It is important to note, however, that the coproduct PPi was not present in the ZmEDS assay with hedycaryol, which could explain the lack of activity. Binding of PPi induces and maintains the appropriate catalytic conformation in TSs. The intramolecular cyclization and protonation of hedycaryol therefore seemingly depends on the exact conformation of the enzyme–substrate complex upon initial FPP binding. This is reminiscent of the result described in section 2.3 where mTSs known to proceed via isomerization sometimes cannot process LPP. The solubility and therefore bioactivity of terpenoids generally relies on the presence of at least two spatially separated oxy groups, which provide the hydrogen-bonding capacity that allows binding to molecular targets.^163^ The discovery and characterization of enzymes capable of directly performing dihydroxylation therefore offers huge potential in multiple industrial and pharmaceutical settings.

## 3 Predictive and High-Throughput Engineering of TSs

Ultimately, the goal is to use the insights described above to make feasible the industrial production of terpenoids or even to create “designer terpene synthases”. In this final section, we discuss how modern computational, experimental, high-throughput (HTP), and machine learning (ML) methods could enable us to reach these goals. These studies seek to overcome the major challenges in TS engineering, in particular, the low sequence identity and the difficulty in characterizing TSs in high-throughput mode.

### 3.1 Systematic Mutagenesis and the Discovery of Plasticity Regions in TSs

**Figure 8:**
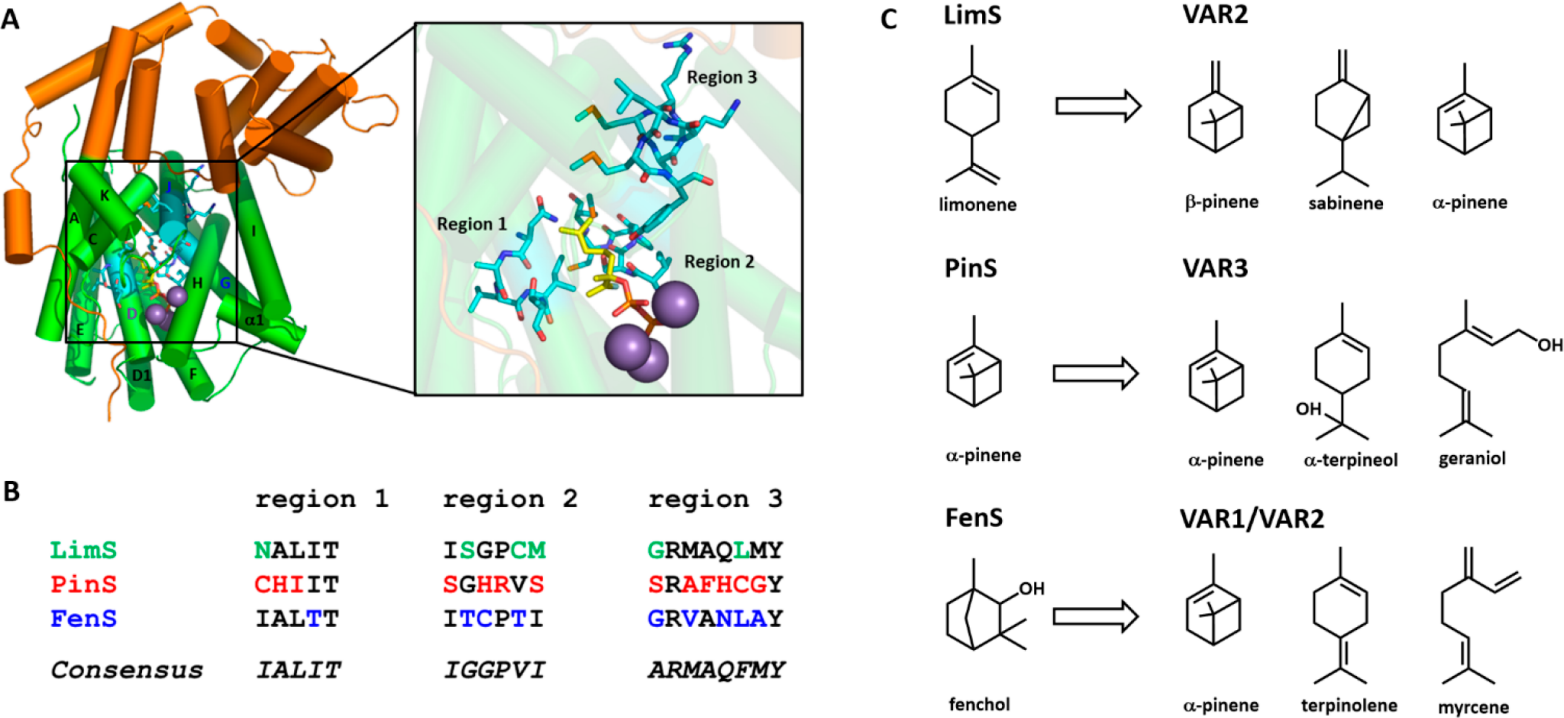
Mutational analysis of plasticity regions in increasingly complex plant mTS cyclization cascades. (A) Identified plasticity regions mapped onto the structure of Ms(−)-LimS (PDB: 2ONG).^52^ The N-terminal domain is colored orange, the C-terminal class I TS domain green, the substrate analogue yellow, and the Mn^2+^ ions in purple. The plasticity regions are highlighted in cyan. (B) WT vs consensus sequences of the targeted enzymes. Each residue targeted by mutagenesis is colored. (C) Main products produced by WT mTSs and key variants. Panels A and B are reprinted with permission from ref (21). Copyright 2018 American Chemical Society.

We have seen how a small number of changes to a TS active site can change its product profile, activity, and substrate specificity. A detailed comparison of several mutational studies on plant mTSs led to the identification of a number of structurally conserved “functional plasticity regions”. The first region is located just upstream of the DDxxD/E metal-binding motif on helix D. A second region covers the G1-G2 helix break motif,^164^ and a third region is located near the C-terminus on helix J (Figure 8).^21,140^ A fourth region has also recently been identified on helix F.^165^ As more mutational studies on plant (and other) TSs become available, it is likely that more functional plasticity regions will be identified.

To see if residues in these regions have universal roles, three mTSs with increasingly complex cyclization cascades were subjected to mutagenesis. These enzymes are Ms(−)-LimS, PtPinS, and fenchol synthase (LvFenS) from *Lavandula viridis*, which make monocyclic, bicyclic, and hydroxylated bicyclic products, respectively (Figure 8). When consensus sequences were introduced in the plasticity regions, exchanges in regions 1 and 2 had the most effect. In most cases, the products detected were the result of premature quenching of the reaction cascade, with both LvFenS variants unable to catalyze the water attack step and both PtPinS variants showing no activity. Interestingly, residue exchanges in region 2 of Ms(−)-LimS resulted in the formation of *more* complex bicyclic products, representing one of only few examples available where products of variant TSs are not the result of premature quenching. Residue exchanges in region 3 had either no effect or only minor effects, shifting the product profiles to a mixture of acyclic, monocyclic, and bicyclic products.^21^ In a follow up study on a promiscuous PtPinS variant, a library with 192 possible variants was screened using an automated pipeline, and several charged residues in regions 1 (H336) and 2 (H445 and R446) were found to be essential for activity, which explains why the consensus variants were inactive. Furthermore, not all residues in the plasticity regions have an equal contribution to overall product outcome, this “power” residing with a few residues within these regions, an important consideration for any rational design strategy.^166^

More systematic mutational studies have been performed on plant TSs, but due to the limited screening capabilities of GC-MS, the gold-standard for TS product analysis, many of these studies have still targeted only a relatively small region of the enzyme. Nonetheless, these systematic approaches have yielded deeper insights into the catalytic and mutagenesis landscapes of TSs. Salmon and co-workers investigated the emergence of cyclization in two sTSs from *A. annua* using structure-based combinatorial protein engineering (SCOPE).^105^ A total of 24 target positions were identified in BFS, including first and second tier residues as well as residues on a flexible loop capping the active site. The library was designed with multiple combinatorial mutations and includes >25,000 theoretically possible mutations. As screening such large number of variants using GC-MS is not feasible, the throughput was increased by screening subsets of discrete variant pools. Several rounds of screening revealed the Y402L variant and the Y402–V467–Y430 epistatic network described in section 2.3. The very existence of these epistatic and coevolution networks in TSs means their fitness landscapes are rugged and discontinuous, facilitating functional change with only a few mutations.^167−169^

### 3.2 Computational Tools for TS Engineering

Many TS studies, such as those described in the 2017 review by Christianson, depend on solved crystal structures; yet despite the numerous known TSs there is only a limited amount of structural data. Homology modeling has been used to predict TSs structures, but while the overall TS fold is highly conserved, correctly predicting enzyme–substrate interactions is much more challenging. The recent development of protein structure prediction algorithms such as AlphaFold can help fill this gap.^170^ Yan and colleagues applied a comprehensive modeling approach—including AlphaFold, molecular docking, MD simulations, and QM/MM calculations—to generate a catalytically active structure for JeSTS4, a class I sTS from the nonseed land plant *Jungermannia exsertifolia*.^171^ JeSTS4 contains a single class I TS domain only, similar to fungal and bacterial TSs, and is part of the family of microbial terpene synthase-like (MTSL) enzymes previously identified in other nonseed plants.^172^ The simulated structure and coevolution analysis revealed two mutational hotspots, with G91S and R242K resulting in improved product yield and catalytic activity compared to the WT.

Another example is the study by Gu and co-workers on 2-methylisoborneol synthase from *S. coelicor* (ScMIBS), which catalyzes the cyclization of 2-methyl-GPP to 2-methylisoborneol.^173,174^ Using phylogenetic analysis, sequence alignments, and available structural data for ScMIBS,^175^ a total of 31 conserved target residues were selected. Site-directed mutagenesis of ScMIBS and structural analysis of interesting variants using AlphaFold resulted in the identification of a conserved salt-bridge important to correct folding and of residues involved with control of active site water molecules.

**Scheme 8:**
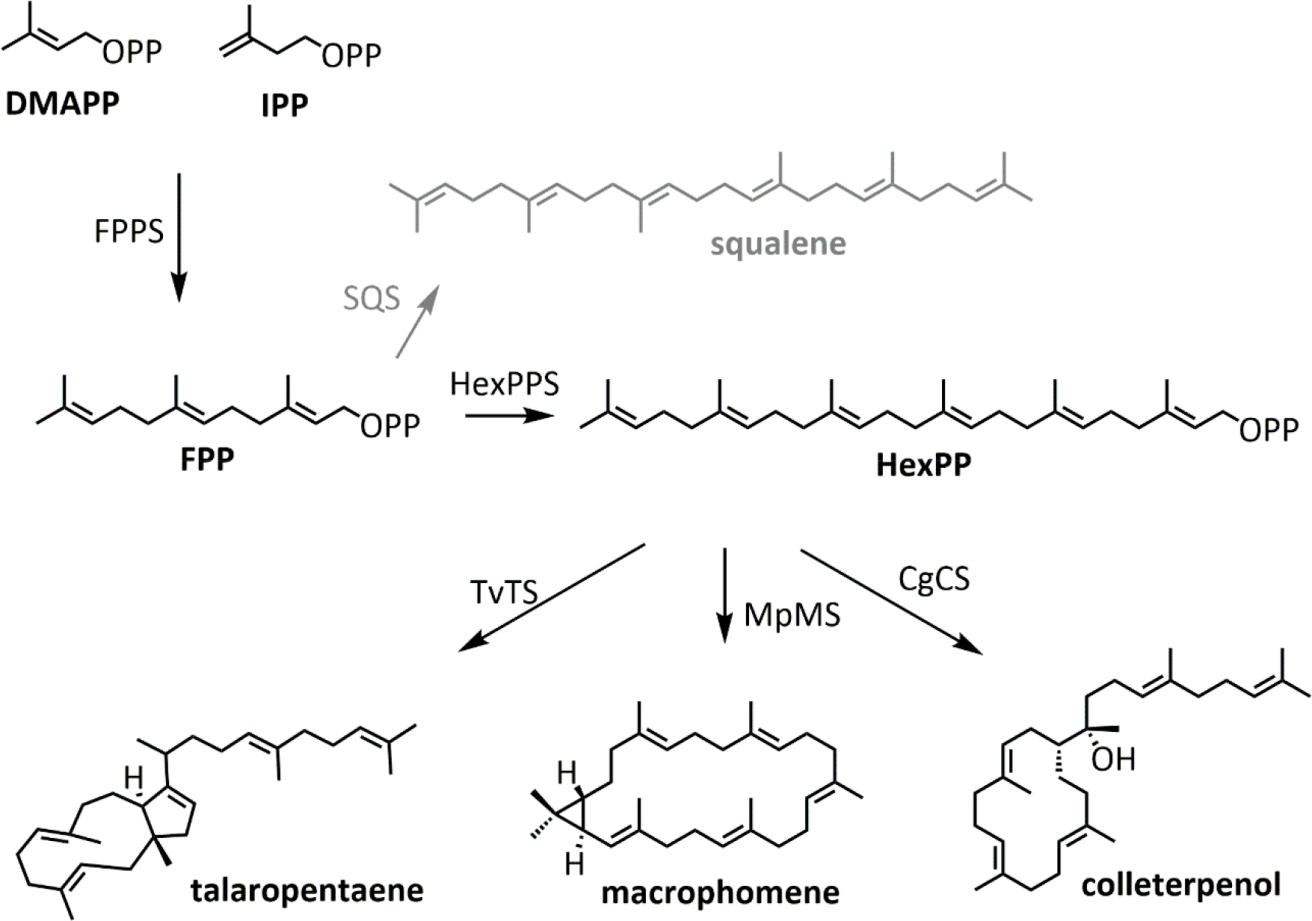
Discovery of Nonsqualene Triterpenoids Triterpenoid biosynthesis typically proceeds through squalene, but three fungal tTSs were recently discovered which proceed via HexPP.

For higher order TSs, two fungal chimeric class I triterpene synthases (tTSs) were recently characterized: *Talaromyces verruculosus* talaropentaene synthase (TvTS) and *Macrophomina phaseolina* macrophomene synthase (MpMS). Comprising prenyltransferase (PT) and terpene synthase/cyclase (TS) domains, these bifunctional enzymes cyclize hexaprenyl diphosphate (HexPP) to give their respective products (Scheme 8). These enzymes, therefore, represent the first examples of nonsqualene-dependent tTSs.^176^ Docking of HexPP reveals that TvTS has a tunnel to accommodate two nonreacting isoprenoid units, consistent with the product talaropentaene possessing a “cycle + tail”, similar to sesquiterpenoids derived from the bisabolyl cation. MpMS, by contrast, contains a larger active site which can accommodate macrocyclization. This was reinforced by MpMS variants (V206F and A207W) that abolished macrophomene production.

The similarity of the solved TvTS crystal structure with the model predicted by Alphafold prompted the authors to develop a screening method for chimeric tTSs based on the differences between TvTS and MpMS, which yielded a novel tTS, *Colletotrichum gloeosporiodes* colleterpenol synthase (CgCS), which follows the same “spectator” reaction as TvTS. Such a discovery would be impossible from sequence analysis alone.

The flexibility of the substrate within the active site has been discussed as a major factor in the product outcome and fidelity in TSs. Consensus docking takes advantage of the intermediate- and product-like binding in TSs and deduces a binding pose based on minimal repositioning between chemical steps along the presumed reaction coordinate.^177^ Examples include TerDockin, designed specifically for TSs, and EnzyDock.^178,179^ Both have been applied successfully to TSs: for example, EnzyDock revealed that the inclusion of an active site water molecule present in the crystal structure of BPPS was crucial for producing a catalytically relevant binding mode for GPP.

### 3.3 High-Throughput Screening for TS Activity

As noted, the limited screening capabilities of GC-MS methods undermines high-throughput (HTP) characterization of TSs. By contrast, data-driven approaches such as machine-learning (ML) can produce models that provide insights into the reaction chemistry of TSs without the need for experimental characterization, but these are very “data-hungry” and require a large number of sequence-function observations to reach acceptable performance levels. This sequence-function data can be obtained from naturally occurring sequence diversity, obtained for example from genome-mining studies^49,180,181^ or directed evolution (DE) experiments, but the latter is reliant on the availability of efficient HTP screening assays. The development of these assays for TS activity is, therefore, a high priority.

Certain enzyme features, for example, thermostability,^182^ have been used as a proxy for activity, while several alternative screening assays for the detection of TS activity have also been developed, including the colorimetric or fluorimetric detection of PPi release using malachite green.^183^ The amount of PPi released is directly proportional to TS activity and can be applied to most TSs, except for those like bornyl diphosphate synthase which recapture the PPi in their product.^56^

Dyes can also be used for the colorimetric detection of terpenoid products. For example, 2,2-diphenyl-1-picrylhydrazyl (DPPH) has been used for the detection of monoterpenoids in a radical-scavenging assay, although the sensitivity of the assay depends on the particular monoterpenoid product.^184^ A HTP screening assay for the detection of microbially produced hydrocarbons, including isoprenoids, was developed using the fluorescent dye Nile red.^185^ In another study, small differences in (*E*)-β-farnesene output were detected with Nile red using a Printed Droplet-Microfluidics platform.^186^ The use of a fluorescent dye in combination with detection in whole cells is ideal, as it opens up the possibility of using truly HTP methods, such as fluorescence activated cell sorting (FACS). Screening performed directly on whole cells or cell extracts expressing the variant TS libraries along with a heterologous isoprenoid production pathway avoids laborious protein purification protocols, and the presence of these heterologous pathways could actually be exploited for HTP screening. An early example couples terpenoid formation to growth, making use of the inherent toxicity of the isoprenoid pyrophosphate precursors. This method was used to enrich a population of engineered *E. coli* with enhanced intracellular FPP levels for clones that express a sTS, and can be used to screen for TS activity.^187^ In another example, terpenoid formation competes with additional artificial pathways for a shared substrate pool where increased TS activity is linked to reduced pigmentation in host cells expressing carotenoid pigments. This change in pigmentation can be detected in colonies coexpressing variant TS genes in carotenoid-producing cells directly on agar plates. As all TSs ultimately use the same isoprenoid substrates, this screening assay is compatible with any TS.^188^ Tuning of the system to elevate the flux from GPP to carotenoid production allowed the screening of a pinene synthase library for improved activity, as discussed in section 2.4.^131^ In another study, intracellular isoprene concentrations are linked to a fluorescence output using a genetically encoded biosensor.^189^ The biosensor was developed to enable HTP screening for improved isoprene production in engineered microbes, but a reduction in the fluorescence output could be used as a probe to screen for enhanced TS activity. The major advantage of these methods is that they can be used on any TS and, since intracellular TS activity is linked to growth or color development, the output can be directly observed in live cells resulting in massively increased throughput compared to conventional GC-MS.

However, none of these methods allow rapid detection of multiple volatile terpenoid compounds, which is essential for TS engineering studies, where altered product outputs are the desired result. Some attempts have been made, however, to distinguish between product properties in the HTP TS screening assays. One such method involves the use of isoprenoid pyrophosphate substrate analogues containing a vinyl methyl ether functional group. Upon cyclization of the modified substrate methanol is released and converted by alcohol oxidase into formaldehyde, which subsequently reacts with a dye to yield a purple color, indicating cyclization and allowing the assay to be performed in 96-well plate format.^190^ In addition to the aforementioned isoprene biosensor, a prokaryotic transcription factor was repurposed as a genetically encoded biosensor for the detection of cyclic monoterpenoids. A DE approach was used to expand the specificity in a camphor-responsive system from *Pseudomonas putida* for the detection of bicyclic monoterpenoids in *E. coli*. Intracellular accumulation of these compounds ultimately results in GFP expression.^191^ This biosensor is a generalist bicyclic monoterpenoid biosensor, but further rounds of evolution may lead to acyclic, monocyclic, or even compound-specific biosensors, paving the way for the ultra-HTP screening of complex terpenoid mixtures.

Many of the TS HTP screening methods described still require GC-MS analysis of variants picked up in the screen and therefore the extraction of the metabolites from liquid cultures. A recent study, however, used supercritical fluid extraction-supercritical fluid chromatography-triple quadrupole mass spectrometry (SFE-SFC-MS/MS), a continuous metabolite extraction and mass analysis method, to directly analyze microbial terpenoid-producing colonies. This is the first example of terpenoid metabolite detection using an analytical technique equivalent to GC-MS directly on microbial colonies without the need for sample pretreatment, reducing the time between library generation and analysis by several days.^192^ In another recent example, proton transfer reaction mass spectrometry (PTR-MS) was used to detect headspace terpenoid concentrations of bacterial cultures expressing *Callitropsis nootkatensis* valencene synthase (CnVS) variants. PTR-MS allows the detection of volatiles in real time, and miniaturization of cultures in 96-deep well blocks allowed peak separation between different samples.^193^ Most HTP TS screening methods result in no to low terpenoid resolution, allowing for the screening of enhanced activity only, whereas high-resolution GC-MS-based techniques often result in much lower throughput (Table 1). Prescreening using a HTP activity screen or ML algorithm combined with a high-resolution method may offer the best results.

**Table 1:** Summary of TS High-Throughput Screening Methods^a^

screening method	detection	sample format	throughput	terpenoid product resolution	reference(s)
Malachite green	Colorimetric detection of PPi release	Purified enzyme in 96-well plates	High	None – Detection of TS activity only	(183)
DPPH radical scavenging	Colorimetric detection of monoterpenoids	Extracted cultures in 96-well plates	High	None – Detection of mTS activity only	(195)
Nile red	Fluorescence detection of intracellular hydrocarbons	Single cells using microfluidics or FACS	Very high	None – Detection of TS activity only	(185,186)
Carotenoid competition screening	Colorimetric colony detection	Microbial colonies	High	None – Detection of TS activity only	(131,188)
Genetically encoded isoprene biosensor	Fluorescent detection of intracellular isoprene concentration	Single cells using microfluidics or FACS	Very high	None – Detection of TS activity only	(189)
Vinyl ether substrate analogues	Colorimetric detection of methanol release upon substrate cyclization	Crude *E. coli* lysate in 96-well plates	High	Low – Cyclic (sesqui)terpenoids	(190)
Automated GC-MS pipeline	Direct detection of microbially produced terpenoid mixtures	Extracted cultures in 96-well plates	Medium	Full product resolution	(166)
Genetically encoded biosensor for cyclic monoterpenoids	Fluorescent detection of intracellular cyclic monoterpenoid concentration	Single cells using microfluidics or FACS	Very high	Bicyclic monoterpenoids	(196)
PTR-MS headspace	Real-time headspace terpenoid detection	Microbial cultures in 96-deep well blocks	High	Low – Selected *m*/*z* values	(193)
SFE-SFC-MS/MS	SFE extracted intracellular terpenoid detection	Microbial colonies	High	Full product resolution	(192)

a Most HTP screening methods result in no to low terpenoid resolution, whereas high-resolution GC-MS-based techniques often result in a much lower sample throughput.

In addition to screening variant libraries to improve TS properties, screening methods have also been developed to discover novel TS activities in metagenomics samples. As discussed elsewhere in this Review, soil bacteria are a rich source of TS activity, and the genomes of many uncultivated organisms remain untapped. Kwak and colleagues developed an HTP functional metagenomics screening method for the identification and isolation of TSs from bacterial metagenomes based on relieving the toxicity of isoprenoid pyrophosphate precursors by functional TSs. Functional screening of human faecal metagenomes resulted in the identification of a novel β-farnesene synthase without amino acid similarity to known class I or class II TSs.^180^ In another example, a set of putative TS gene fragments with similarity to the known bacterial TSs geosmin synthase and 2-MIBS were detected in environmental DNA extracted from a subseafloor sediment sample.^194^

### 3.4 Data-Driven Approaches

As discussed previously, rugged fitness landscapes and epistatic networks allow for functional change with only a few amino acid substitutions. Within the context of TS engineering toward “designer” product profiles, this offers huge potential, but identifying and predicting the effects of these networks is labor-intensive. The application of data-driven methods such as machine learning (ML) can help navigate the vast sequence spaces and rugged fitness landscapes which arise due to nonlinear phenomena such as coevolution and epistasis.^167^

But rather than looking at individual enzymes, data-driven approaches such as ML are increasingly being used to assist protein engineering experiments by learning from the properties of characterized variants to predict variants with improved properties.^197^ Additionally, as ML models can be used to virtually screen a large number of sequences, the screening in a DE experiment is significantly reduced.^198^ The need for large amounts of input data has meant that ML in protein engineering has been dominated by sequence-based methods. However, with the development of tools such as AlphaFold, structural information is now being used as input and contains more information than amino acid sequences alone.^199^ A structure-based approach is particularly relevant to TSs where protein sequences can be diverse, but the overall fold is highly conserved. One of the few available example studies of ML applied to TSs combined different computational approaches to predict precursor cation specificity for plant sTSs.^143^ Selection for either the farnesyl or nerolidyl cation is a fundamental step in sTS-catalyzed reactions and is determined early on in the reaction cascade (Scheme 3). Using a structure-based ML approach it was possible to predict farnesyl or nerolidyl cation specificity accurately for class I plant sTSs, and a correlated mutation analysis on the identified cation-specific residues on thousands of uncharacterized, putative plant sTS sequences resulted in the identification of coevolved cation-specific residue pairs. These are located in five structural regions which, gratifyingly, partly overlap with the plasticity regions identified in plant mTSs.^21^ As more characterized TSs become available, the models will get more accurate and the predictions more specific, including the prediction of certain cyclization events or even final products. Furthermore, it has been shown that multiomics data analysis, including genomic, transcriptomic, volatilomic, and metabolomics data, can predict the functions of putative TSs. Even though fungi, like bacteria, are increasingly recognized as valuable sources for TS activity,^200^ far fewer have been characterized compared to plants, providing insufficient data to perform ML studies. Using an alternative multiomics approach, Nosenko and co-workers predicted the product profiles for 116 out of 146 putative sTS genes from 30 different fungal species.^201^ The prediction method is based on the assumption that closely related sTS genes likely share the same initial steps in the reaction cascade, even though the final products may be quite divergent, a feature that was also observed for plant sTSs.^61^ Combined with gene expression information and patterns of volatile compound emission, this allowed the authors to assign function to the putative fungal sTSs. This kind of multidisciplinary approach, which integrates sequence diversity data, genome mining studies, HTP product profiling, modeling, and computational chemistry in data-driven ML models, will enable the engineering of designer TS activities in the near future.^92^

## 4 Conclusion

Terpenoids are valuable compounds that are found in a variety of established and emerging industries. TSs, the enzymes responsible for producing terpenoids, demonstrate a level of stereo- and regiochemical control far beyond that of chemical synthesis and can turn simple linear substrates into thousands of different terpenoid compounds, making them highly attractive engineering targets. The perilous reaction coordinate, with its myriad competing pathways, means TSs have evolved to prioritize chemical control over efficiency. Indeed, the only rate-enhancing step played by TSs might be reaction initiation. The active sites of TSs are carefully sculpted to achieve a particular outcome, which depends in large part on the binding mode and subsequent flexibility of the substrate. As such, the product profile of many TSs can be altered with very few amino acid changes, and a correlation between the fidelity and active site composition has begun to emerge. This plasticity has implications for the evolution of TSs and can be harnessed for their rational design. It has been noted that plant TSs typically demonstrate greater plasticity than their bacterial and fungal analogues, with these latter enzymes usually showing greater fidelity. The untapped potential of these organisms will surely be important in the near future of TS engineering. Despite all of this, TSs have inherent limitations. Structure–function relationships are hard-earned, and a low degree of sequence identity means insights from one TS are not always applicable to another. The catalytic motifs described in this Review represent a move toward a more general understanding of TS chemistry. Computational and data-driven techniques are overcoming the lack of sequence identity, and recent innovations such as AlphaFold are negating the need for structure determination all together. Modern experimental and data-driven techniques also present the opportunity to interrogate and engineer TSs in high-throughput mode, substantially increasing the speed at which new insights can be obtained. This kind of multidisciplinary approach will move us closer to the ultimate goal of TS engineering, designer terpene synthases.
